# PPIH acts as a potential predictive biomarker for patients with common solid tumors

**DOI:** 10.1186/s12885-024-12446-9

**Published:** 2024-06-04

**Authors:** Jun Ye, Jianchao Ying, Haixia Chen, Zhiping Wu, Chaolin Huang, Chuan Zhang, Zhitao Chen, Haini Chen

**Affiliations:** 1 Department of Clinical Laboratory, The Second Affiliated Hospital of Guizhou Medical University, Kaili, Guizhou, 556000 China; 2 Wenzhou Key Laboratory of Emergency, Critical Care, and Disaster Medicine, Central Laboratory, The First Affiliated Hospital of Wenzhou Medical University, Wenzhou, China; 3 College of Laboratory Medicine, Guizhou Medical University, Guiyang, Guizhou, 550001 China; 4 Department of Gynaecology, The First Affiliated Hospital of Chengdu Medical College, Chengdu, China; 5 Department of Pathology, The Second Affiliated Hospital of Guizhou Medical University, Kaili, Guizhou 556000 China

**Keywords:** *PPIH*, Common solid tumors, Predictive biomarker, Combined diagnosis, TCGA

## Abstract

**Background:**

Our previous studies have indicated that mRNA and protein levels of *PPIH* are significantly upregulated in Hepatocellular Carcinoma (LIHC) and could act as predictive biomarkers for patients with LIHC. Nonetheless, the expression and implications of *PPIH* in the etiology and progression of common solid tumors have yet to be explored, including its potential as a serum tumor marker.

**Methods:**

We employed bioinformatics analyses, augmented with clinical sample evaluations, to investigate the mRNA and protein expression and gene regulation networks of *PPIH* in various solid tumors. We also assessed the association between *PPIH* expression and overall survival (OS) in cancer patients using Kaplan-Meier analysis with TCGA database information. Furthermore, we evaluated the feasibility and diagnostic efficacy of PPIH as a serum marker by integrating serological studies with established clinical tumor markers.

**Results:**

Through pan-cancer analysis, we found that the expression levels of *PPIH* mRNA in multiple tumors were significantly different from those in normal tissues. This study is the first to report that *PPIH* mRNA and protein levels are markedly elevated in LIHC, Colon adenocarcinoma (COAD), and Breast cancer (BC), and are associated with a worse prognosis in these cancer patients. Conversely, serum PPIH levels are decreased in patients with these tumors (LIHC, COAD, BC, gastric cancer), and when combined with traditional tumor markers, offer enhanced sensitivity and specificity for diagnosis.

**Conclusion:**

Our findings propose that *PPIH* may serve as a valuable predictive biomarker in tumor patients, and its secreted protein could be a potential serum marker, providing insights into the role of *PPIH* in cancer development and progression.

**Supplementary Information:**

The online version contains supplementary material available at 10.1186/s12885-024-12446-9.

## Introduction

Cancer continues to be a principal cause of mortality globally, inflicting profound distress on individuals and defying effective prevention and treatment [1]. Despite relentless efforts yielding enhanced insight into tumor pathogenesis and the evolution of treatments from radiotherapy and chemotherapy to immune checkpoint (ICP) blockade and targeted drug therapies, challenges persist. These include limited patient benefits, suboptimal survival rates, and difficulties in achieving lasting therapeutic responses [2–4]. The prognosis for malignant tumors remains grim, partly due to delayed diagnosis and the absence of early, sensitive diagnostic and prognostic markers. Thus, identifying novel therapeutic targets and sensitive tumor biomarkers for early detection and treatment is imperative [5].

*PPIH*, part of the Cyclophilins (Cyps) family and known in prior studies as Cyp-H, USA-Cyp, or U4/U6-20 K [6, 7], is recognized not only for its role in protein folding but also for its significant involvement in pre-mRNA splicing and the U4/U5/U6 tri-snRNP complex assembly [7–9]. Research by Li et al. [10] has associated increased *PPIH* expression in gastric adenocarcinoma (STAD) with poor patient outcomes, whereas *PPIH* downregulation may inhibit STAD cell proliferation, migration, and invasion. Gao et al. [11] confirmed that *PPIH*, as a COVID-19 susceptibility gene in LUAD patients, can increase the susceptibility of LUAD patients to COVID-19 through a variety of biological processes and pathways. Li et al. [12] also highlighted the pivotal role of RNA-binding proteins, including *PPIH*, in the development of HBV-associated hepatocellular carcinoma. Our prior research identified a substantial overexpression of *PPIH* mRNA and proteins in LIHC [13], correlating with advanced disease stages, poor differentiation, and TP-53 mutations. These findings propose *PPIH* as a potential predictive biomarker for LIHC, warranting further exploration of its role in cancer progression. Nevertheless, comprehensive analyses of *PPIH* ‘s gene regulatory network and its diagnostic value in LIHC, as well as its expression in COAD, and BC, remain uncharted territories, making it vital to ascertain *PPIH* ‘s expression profiles and their implications for diagnosis and prognosis in common solid tumors.

Building on previous results linking poor LIHC prognosis to elevated *PPIH* levels, this study aims to delve deeper into *PPIH* ‘s potential function and prognostic significance in common solid tumors. Through bioinformatics analysis utilizing open-access databases, we explored differences in *PPIH* mRNA expression in pan-cancer, and subsequently we meticulously examined *PPIH* expression in LIHC, COAD, and BC, assessing its correlation with patient outcomes. This was complemented by empirical validations such as protein immunoassays and immunohistochemistry (IHC), confirming *PPIH* upregulation in tumor tissues. Additional investigations into *PPIH* ‘s involvement in tumorigenesis were conducted using TCGA, GeneMANIA, and GSEA data sources. Moreover, by integrating serological studies with conventional tumor markers for a joint diagnostic approach, we evaluated PPIH’s potential as a serological marker for solid tumors.

The outcomes of this study may enhance our comprehension of *PPIH*’s functionality in the ontogeny and progression of common solid tumors, positioning *PPIH* as a promising novel prognostic marker and a target for therapeutic interventions.

## Materials and methods

### Patients and LIHC tissue specimens

At the First Affiliated Hospital of Wenzhou Medical University, six pairs of matched LIHC tumor tissue and adjacent normal tissue were collected immediately after surgical resection and instantly quenched in liquid nitrogen. Each pair was obtained from a patient clinically and pathologically confirmed with liver cancer. The Board of Directors and Ethics Committee of the hospital approved the informed consent process, and written informed consent was obtained from all participants.

### Clinical samples and IHC staining

Chengdu GaoxinDaan Medical Laboratory Co., Ltd. provided paraffin-embedded tissue samples of LIHC, COAD, and BC patients for immunohistochemical analysis. Comprehensive clinical data and laboratory results were followed up for all patients, who gave informed consent for participation. We made minor modifications to the previously published IHC staining method [14]. Paraffin sections were incubated overnight at 4°C with an anti-PPIH primary antibody (diluted 1:100), with PBS serving as a negative control and tonsil tissue as a positive control. The sections were then treated with biotin-labeled goat anti-rabbit IgG, followed by the streptavidin-biotin-peroxidase complex at room temperature for 30 minutes. IHC staining utilized 3,3’-diaminobenzidine tetrahydrochloride, with hematoxylin as a counterstain. After ethanol dehydration and xylene washing, samples were mounted. The expression of PPIH protein was evaluated semi-quantitatively, Positive expression was indicated by cytoplasmic staining in yellow or brownish-yellow. Expression intensity scores ranged from 0 to 3, indicating negative (no positive staining), weakly staining (light yellow), moderately staining (brownish-yellow cytoplasm), and strongly staining (yellowish-brown or granular).

### Clinical serum specimens

From September to October 2023, serum samples from sixteen patients each with LIHC, COAD, GC, and BC were collected at the Second Affiliated Hospital of Guizhou Medical University. Inclusion criteria included no history of other malignancies, histologically confirmed solid tumors, and comprehensive clinical and follow-up data. Exclusion criteria encompassed recent systemic infections, autoimmune or active immune diseases, severe hypertension or cardiac conditions, and pregnancy. Control serum samples were also taken from sixteen health check participants. All patients provided informed consent, and the hospital’s Ethics Committee approved the study protocol.

### Western blot analysis

Clinical tissues and whole-cell proteins were lysed with 1% Triton X-100 buffer containing protease and phosphatase inhibitors. The protein concentration was determined using BCA protein assay kits (Beyotime, Shanghai, China). We loaded 40 mg of total protein per sample on 12% SDS-PAGE gels for separation, then transferred onto PVDF membranes (Bio-Rad, Hercules, CA) using a wet transfer system (Bio-Rad, United States). After the blot was sealed in TBST with 5% skim milk at room temperature for 1 h, the membrane segment where the target proteins (PPIH: 19 kDa and β-actin: 42 kDa) were located was cut off according to the molecular weight of Marker. Overnight incubation was performed with specific primary antibodies PPIH (ab235595) (Abcam, Cambridge, MA) (1:2000) and β-actin (Beyotime, Shanghai, China) (1:5000) at 4℃, respectively. After washing with TBST, blots were incubated with HRP-conjugated secondary antibodies and visualized using ECL (Thermo Fisher Scientific, Rockford, IL). ImageJ software quantified the optical density.

### Bioinformatics analysis

TCGA, LinkedOmics, and GeneMANIA databases provided multi-omics information and clinical data for analyzing *PPIH* mRNA expression and prognosis in solid tumors, as well as a comprehensive gene regulatory network analysis for *PPIH* in BC, and LIHC. All database analyses followed previously described methods [14, 15].

### Elisa analysis

The ELISA kit (Jingmei Biological Technology Co., Ltd, Jiangsu, China) instructions were adhered to as follows: (1) Standard product addition and dilution; (2) Test sample addition to designated wells; (3) Incubation at 37℃ for 30 min; (4) Plate washing four times; (5) Enzyme reagent addition; (6) Further incubation and washing; (7) Color development; (8) Reaction termination; (9) Measurement by setting blank well readings to zero, recording absorbance (OD value) at 450 nm wavelength with an enzyme-linked immunosorbent assay reader, and calculating PPIH concentration from the standard curve.

### Statistical analysis

GraphPad Prism (v.9.0) and SPSS 27.0 conducted the statistical analyses, with *p*-values less than 0.05 denoting significance. Kaplan-Meier survival analysis utilized the log-rank test, while Logistic regression and ROC curve analysis were applied for ROC analysis. Two-group comparisons used Student’s t-test.

## Results

### Elevated expression of *PPIH* in three types of cancers

**Fig. 1:**
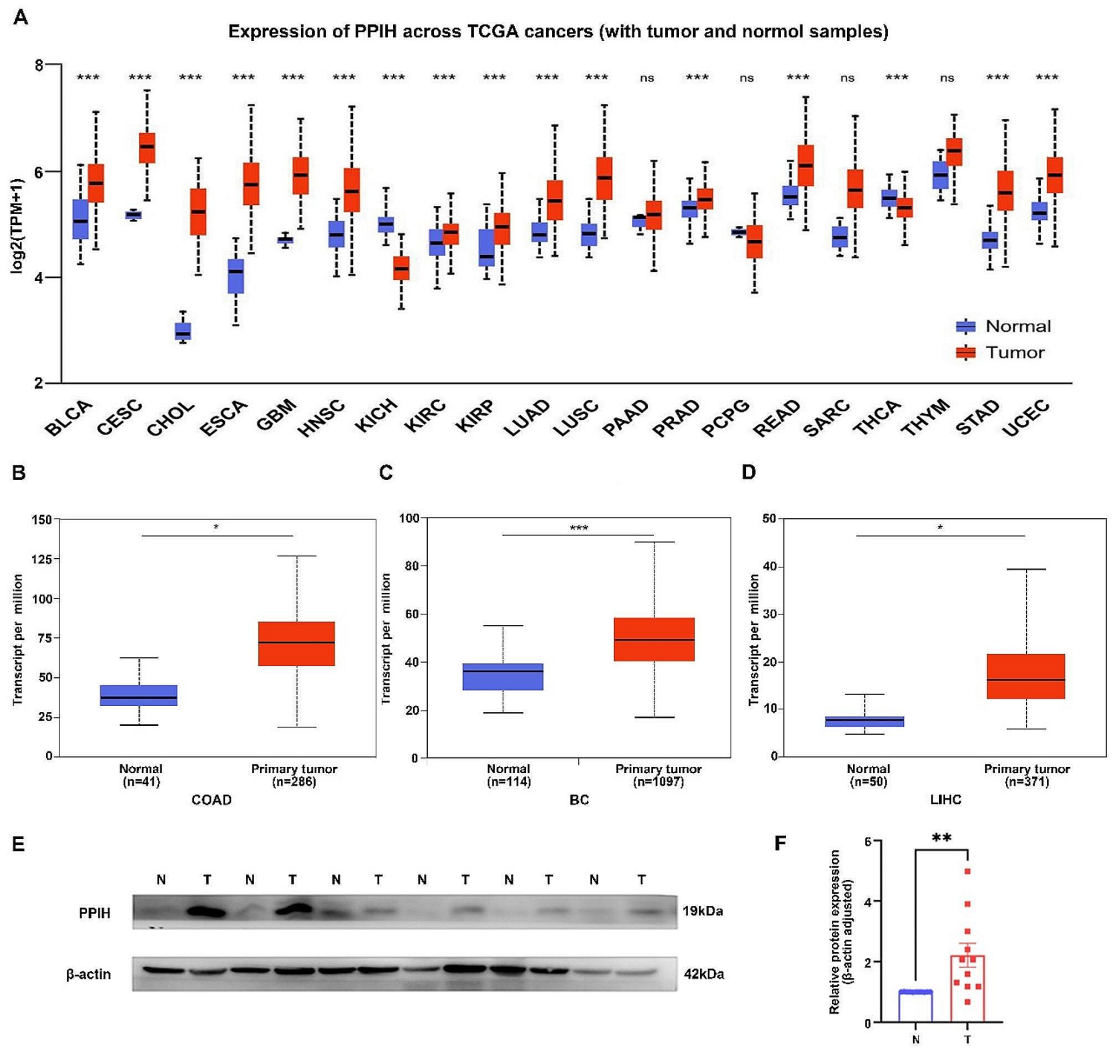
Expression levels of *PPIH* in three types of cancer. **(A)** Comparison of *PPIH* expression between tumor and normal samples(TCGA database). (B) mRNA expression levels of *PPIH* in colon adenocarcinoma tissues versus adjacent normal colon tissues (TCGA database). **(C)** mRNA expression levels of *PPIH* in breast cancer tissues versus adjacent normal breast tissues (TCGA database). **(D)** mRNA expression levels of *PPIH* in Liver cancer tissue and adjacent normal liver tissues (TCGA database). **(E, F)** Representative western blots showing PPIH protein expression in LIHC tissues (T) compared to matched normal liver tissues (N) (*n* = 6). *p < 0.05; **p < 0.01; ***p < 0.001

By analyzing the TCGA dataset, we found that the expression of *PPIH* in cancer tissues was significantly different from that in normal tissues (Fig. 1A). It can be seen from the results that, except for a few tumors such as KICH and THCA, the expression level of *PPIH* is generally elevated in cancer cell lines of different tissue sources. Subsequently, we used TCGA data sources to focus on the differential expression of *PPIH* in COAD, BC, and LIHC. We observed overexpression of *PPIH* in colon adenocarcinoma tissues relative to normal tissues (Fig. 1B). Boxplot analyses revealed that breast cancer specimens exhibited significantly higher *PPIH* mRNA expression than normal tissues (Fig. 1C). Similarly, *PPIH* mRNA expression was elevated in liver cancer tissues compared to adjacent normal liver tissues (Fig. 1D). Furthermore, protein analysis in six LIHC patients (comprising six tumor tissues and six matched adjacent normal tissues) confirmed statistically significant higher levels of PPIH in LIHC tissues (Fig. 1E-F).

### IHC verification of PPIH overexpression in three types of cancers

**Fig. 2:**
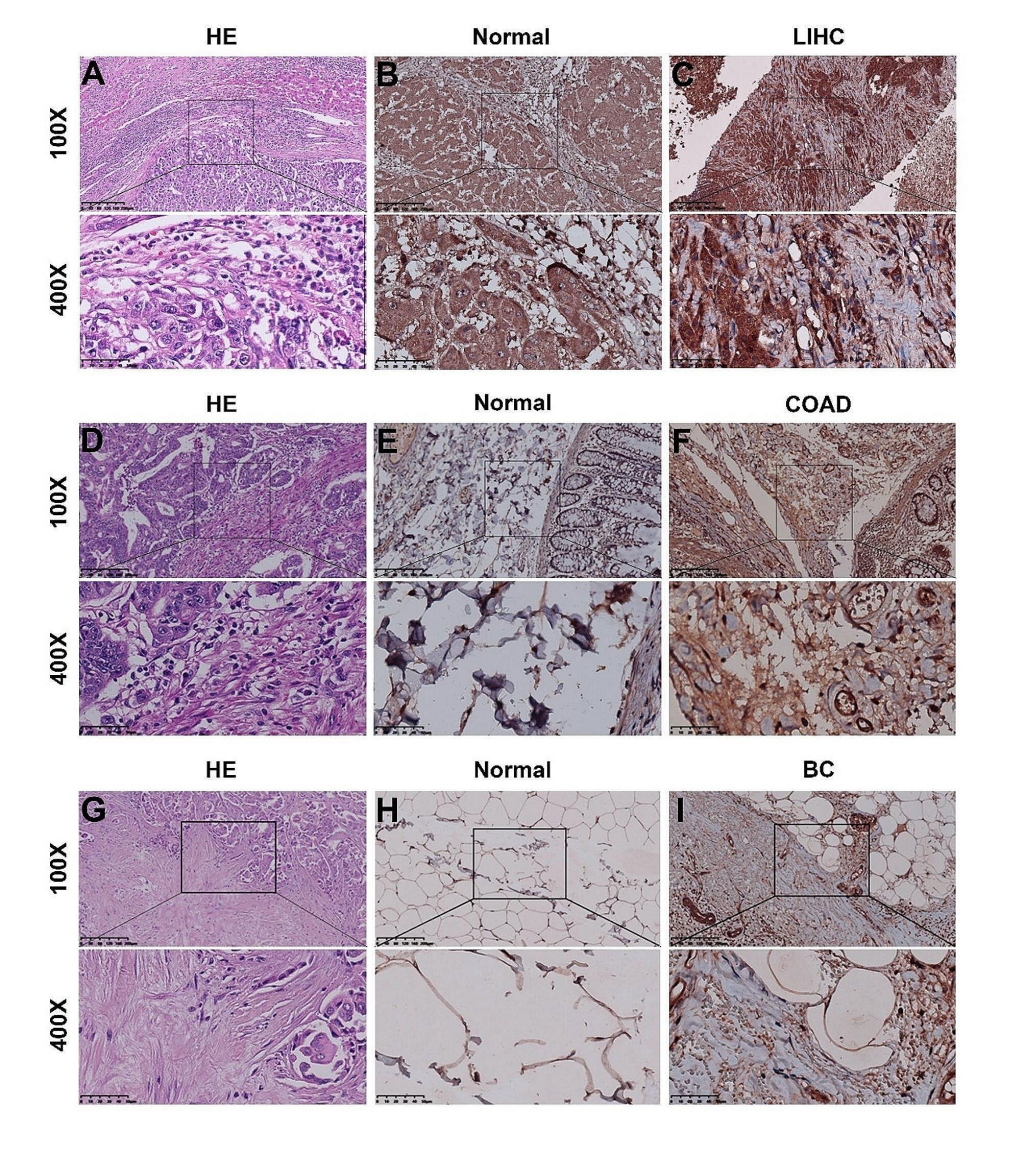
IHC analysis of PPIH for three types of cancers. **(A, D, G)** HE staining of LIHC, COAD, BC. **(B, C)** IHC results showing PPIH levels in LIHC tissues versus normal tissues. **(E, F)** IHC results showing PPIH levels in COAD tissues versus normal tissues. **(H, I)** IHC results showing PPIH levels in BC tissues versus normal tissues

IHC staining was conducted on paraffin-embedded tumor tissues to determine PPIH protein levels. According to the set of semi-quantitative assessment criteria, PPIH staining was moderate in normal liver (Fig. 2A-C), while PPIH staining was strong in tumor tissue. Normal colon tissue (Fig. 2D-F) and normal breast tissue (Fig. 2G-I) showed weak PPIH staining, while tumor tissue showed moderate PPIH staining. These findings collectively indicate a significant overexpression of PPIH in COAD, BC, and LIHC tissues compared to their normal counterparts.

### Prognostic implications of *PPIH* mRNA expression in cancer patients

**Fig. 3:**
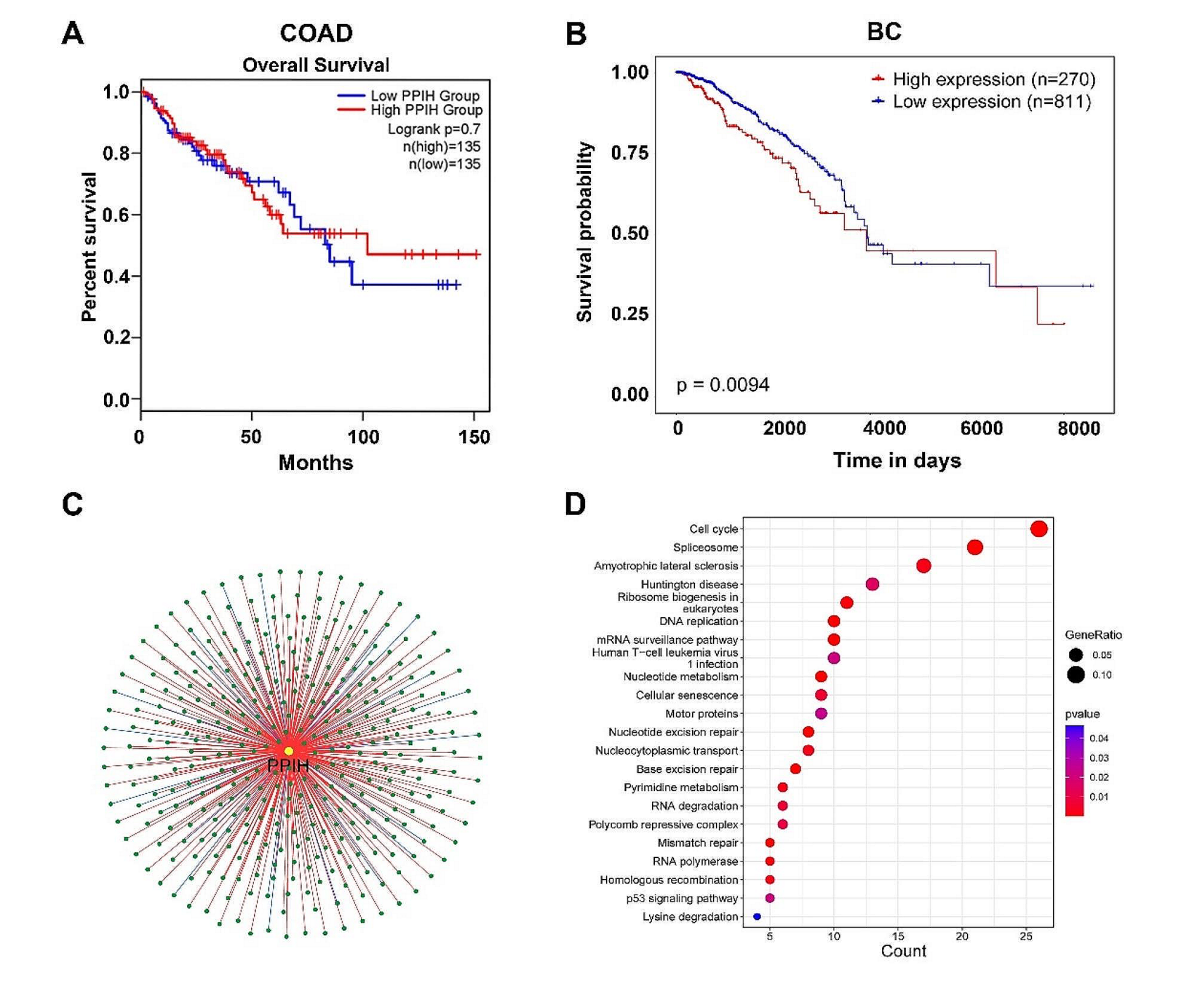
Prognostic significance of *PPIH* in three types of cancer and its biological interaction network in BC. **(A, B)** Kaplan-Meier plots illustrating the correlation between *PPIH* gene expression and survival in patients with COAD and BC (TCGA database). **(C)** Network diagram of the *PPIH* gene and 367 co-expressed genes, with red lines indicating positive correlations and blue lines indicating negative correlations (TCGA database). **(D)** KEGG enrichment analysis of *PPIH* co-expressed genes highlighting pathways with a *p*-value less than 0.05

Previous studies by our team indicated that elevated *PPIH* expression was associated with poor overall survival, progression-free interval, disease-free interval, and disease-specific survival in LIHC patients [13]. In this study, Utilizing the TCGA database, we explored the association between *PPIH* expression levels and oncogenic or suppressive gene activity in relation to patient prognosis in COAD, and BC. Figure 3A and B show that higher *PPIH* expression corresponded with poorer overall survival in BC patients, a statistically significant difference (*P* = 0.0094). However, for COAD patients, Kaplan-Meier survival analysis suggested that high *PPIH* expression did not significantly correlate with a worse prognosis, potentially due to the limited sample size.

### Enrichment analysis of *PPIH* functional networks in cancers

#### KEGG pathway analyses of co‑expression genes correlated with *PPIH* in Breast cancer

Utilizing TCGA mRNA sequencing data, we constructed a network diagram illustrating 367 genes co-expressed with *PPIH* in breast cancer. In this diagram, red lines indicate positive correlations and blue lines negative ones (Fig. 3C). These genes were subjected to KEGG enrichment analysis with an FDR cutoff set at < 0.05. The analysis revealed that the co-expressed genes, mostly associated with *PPIH* in breast cancer, were significantly involved in the cell cycle and spliceosome pathways (Fig. 3D).

**Fig. 4:**
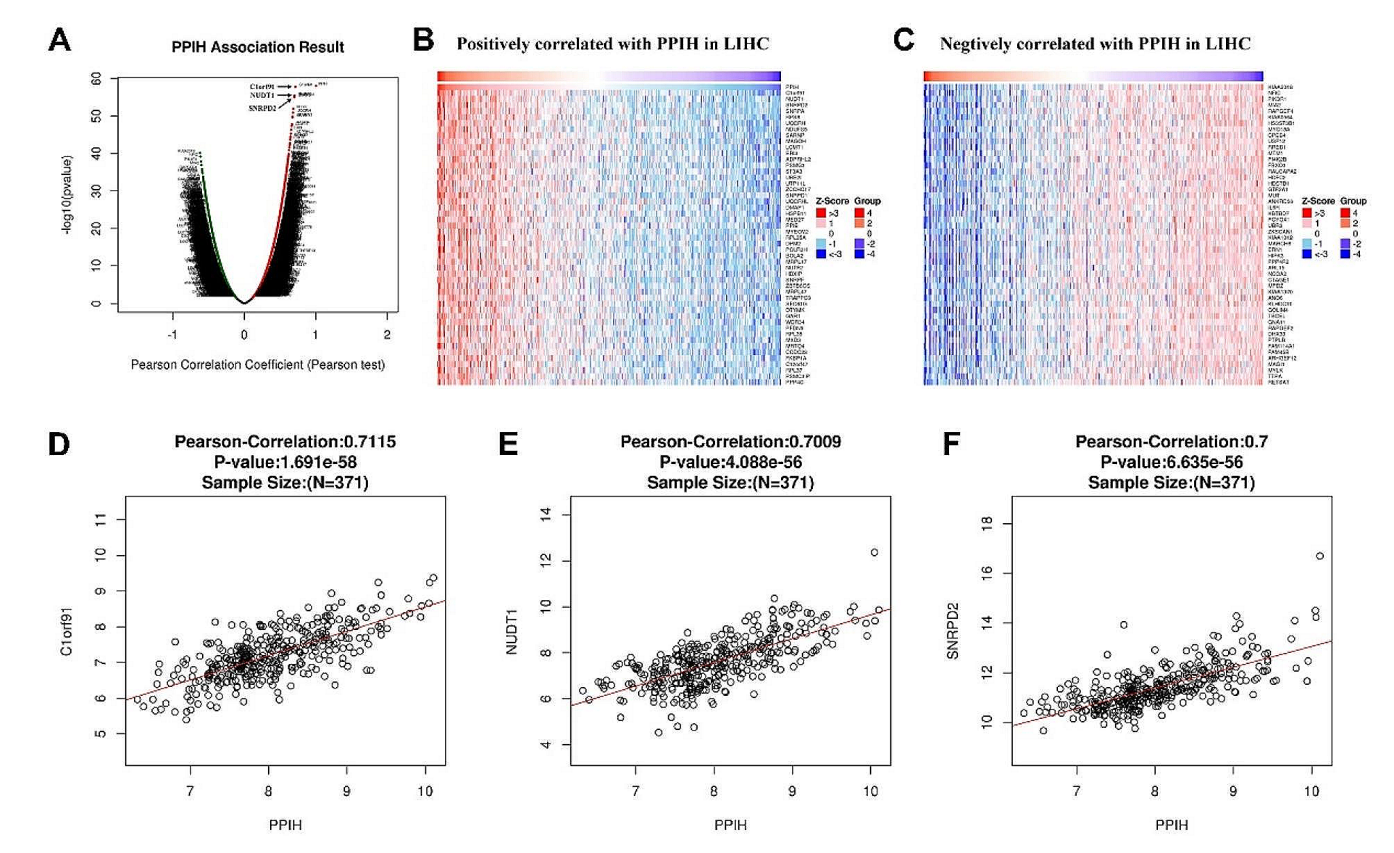
Genes differentially expressed in correlation with *PPIH* in LIHC (LinkedOmics database). **(A)** A Pearson test identified correlations between *PPIH* and differentially expressed genes in LIHC, Red indicates positive correlation and green indicates negative correlation. we selected genes with a correlation coefficient greater than 0.1 or less than − 0.1, respectively, with a false discovery rate (FDR) < 0.01. **(B-C)** Heatmaps display genes with positive and negative correlations with *PPIH* in LIHC (Top 50). Red signifies positive correlations, and blue signifies negative correlations. **(D-F)** Scatter plots confirm the positive correlation between *PPIH* expression and the expression of C1orf91 (D), NUDT1 (E), and SNRPD2 (F)

#### GO and KEGG pathway analyses of co‑expression genes correlated with *PPIH* in LIHC

We employed LinkedOmics to analyze the mRNA sequencing data from 371 LIHC patients within the TCGA database, using a Pearson test to examine the co-expression relationship with *PPIH* (Fig. 4A). Heatmaps display the top 50 genes that showed significant positive or negative correlation with *PPIH* (Fig. 4B-C). As shown in Fig. 4D-F, the mRNA expression of *PPIH* showed the strongest positive association with expression of C1orf91 (Pearson correlation = 0.71, *p* = 1.691e-58), NUDT1 (Pearson correlation = 0.70, *p* = 4.088e-56), and SNRPD2 (Pearson correlation = 0.70, *p* = 6.635e-56), which reflect changes in the cell cycle, RNA polymerase II [16],and spliceosomal components [17].

**Fig. 5:**
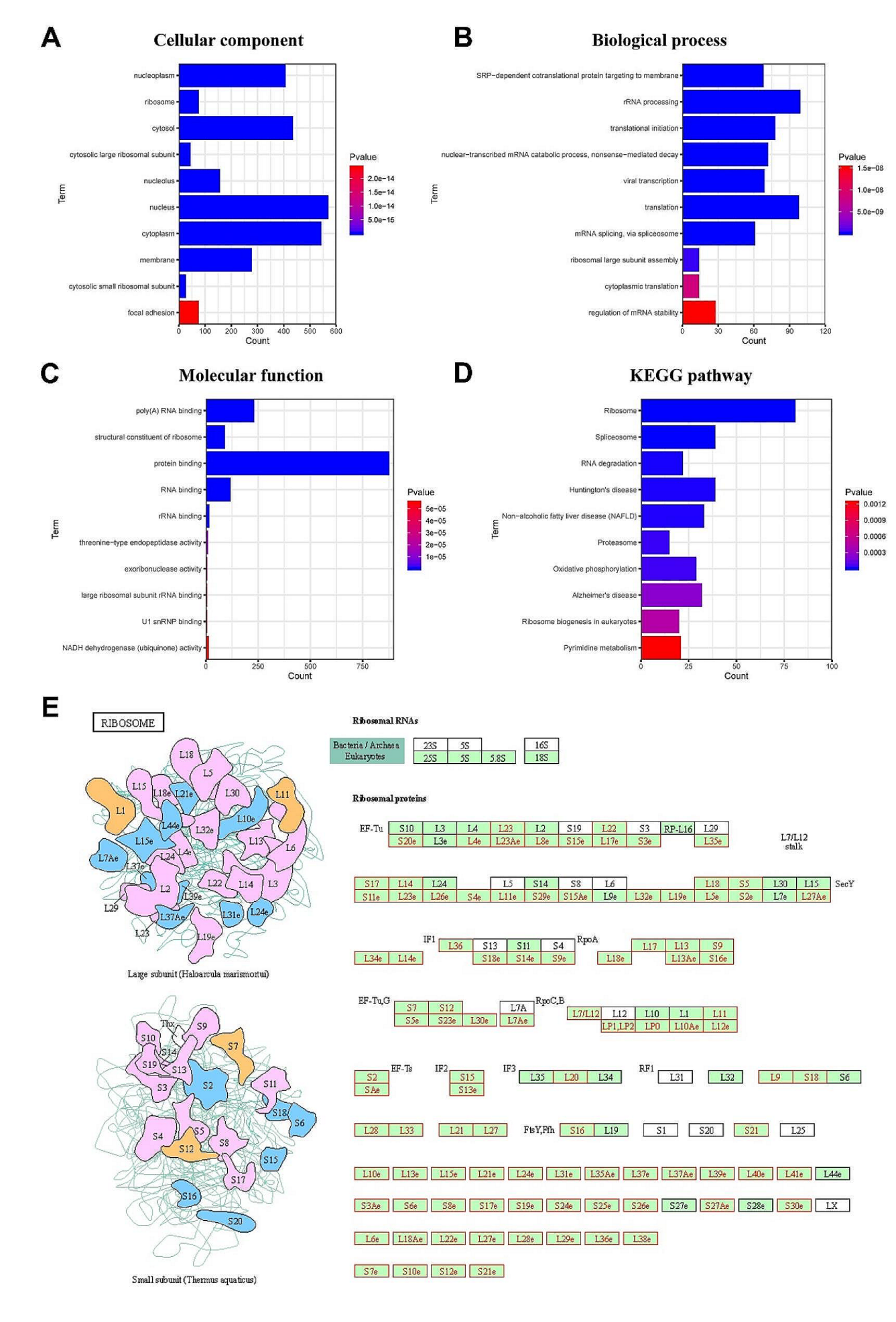
Functional analysis of *PPIH* co-expressed genes in LIHC. **(A-D)** GO analysis and KEGG enrichment for 1626 genes co-expressed with *PPIH* in LIHC. Using the Pearson test (Fig. 4A-C), we selected genes with a correlation coefficient greater than 0.4 or less than − 0.4 (LinkedOmics, DAVID, and bioinformatics databases). **(E)** KEGG pathway annotations for the ribosome pathway, with nodes associated with Leading Edge Genes marked in red. An FDR < 0.05 was considered statistically significant

These significant genes were further analyzed for GO and KEGG enrichment using the DAVID database (Supplementary Tables 1–4), maintaining the FDR cutoff at < 0.05. Cellular component analysis located these proteins primarily in the nucleoplasm, ribosome, and cytosol (Fig. 5A-D). Biological processes were notably enriched in SRP-dependent cotranslational protein targeting to the membrane, rRNA processing, translational initiation, and translation. Molecular function analysis identified significant involvement in poly(A) RNA binding, ribosome structure, protein binding, and RNA binding. The KEGG pathway results indicated predominant participation in the ribosome, spliceosome, RNA degradation, and non-alcoholic fatty liver disease (NAFLD) pathways (Fig. 5E).

#### *PPIH* networks of miRNA and transcription factor targets in LIHC

**Fig. 6:**
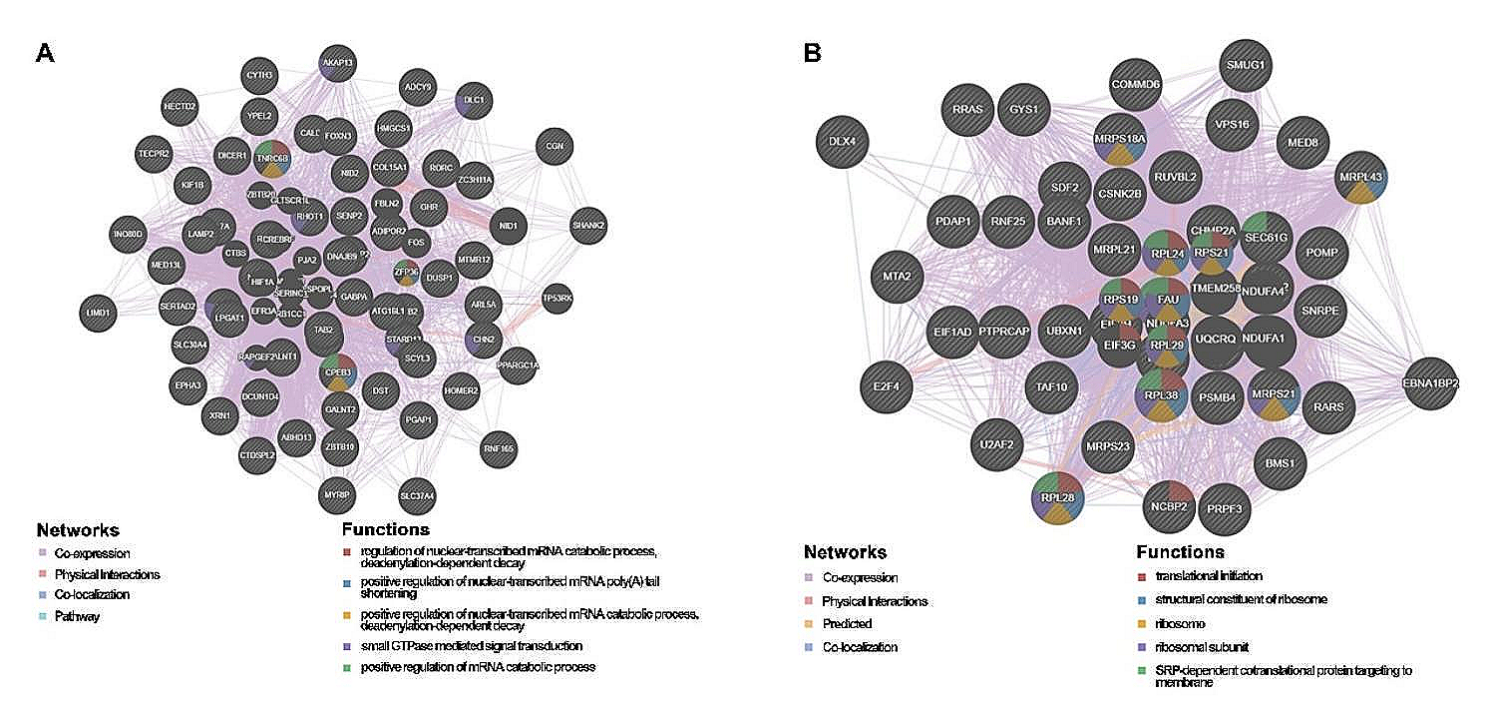
Networks of miRNA and transcription factor targets related to *PPIH* in LIHC (GeneMANIA database). **(A)** Protein-protein interaction (PPI) network for MIR-202. **(B)** PPI network for the transcription factor GGAANCGGAANY_UNKNOWN. Network edges are color-coded to denote the bioinformatics methods used: co-expression, physical interactions, co-localization, and pathways. Node colors differentiate the biological functions of the enrichment gene sets

Further investigation into *PPIH* targets in LIHC identified the top five significant *PPIH*-related genes in the miRNA and transcription factor target networks (Supplementary Tables S5-S6). Notable targets included ATACCTC, MIR-202, and GGAANCGGAANY_UNKNOWN. We then constructed the PPI network for MIR-202 and transcription factor GGAANCGGAANY_UNKNOWN using the GeneMANIA database. This network suggested that miR-202-associated gene sets were mainly implicated in regulating nuclear mRNA catabolic processes, including deadenylation-dependent decay and positive regulation of mRNA catabolic processes (Fig. 6A). Similarly, gene sets connected to the transcription factor GGAANCGGAANY_UNKNOWN were primarily involved in translation initiation, ribosomal structure, and SRP-dependent cotranslational protein targeting to the membrane (Fig. 6B).

### Analysis of PPIH as a diagnostic serum tumor marker

#### Serum PPIH levels are down-regulated in cancer patients

**Fig. 7:**
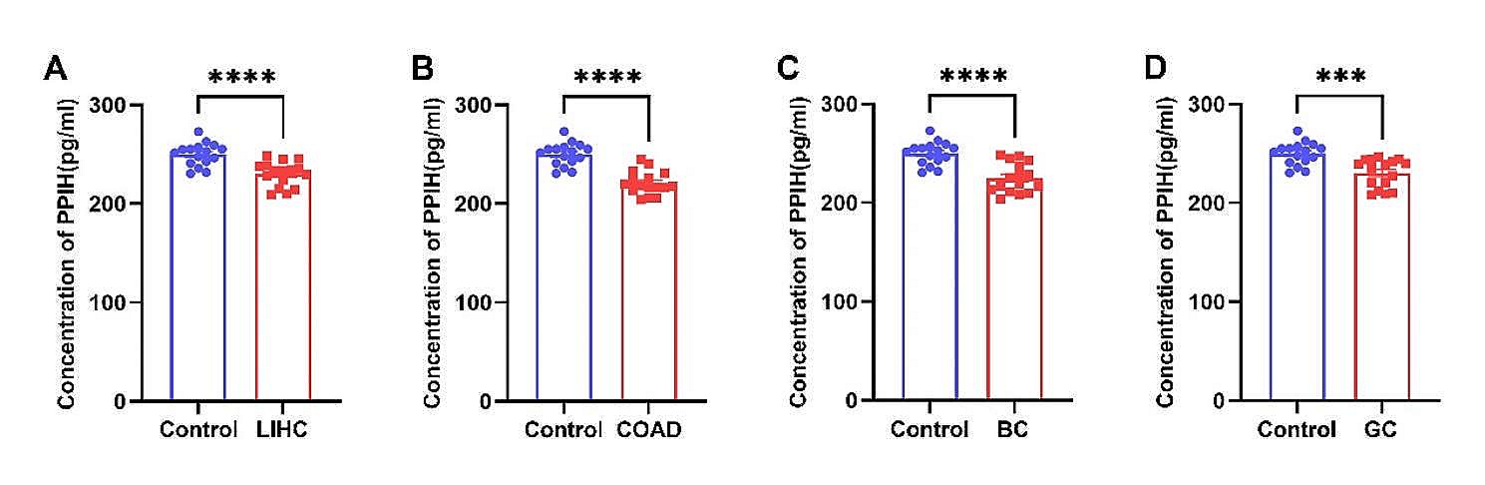
Serum PPIH levels in four cancer types. **(A)** Comparison of serum PPIH levels between LIHC patients and healthy individuals. **(B)** Comparison of serum PPIH levels between COAD patients and healthy individuals. **(C)** Comparison of serum PPIH levels between BC patients and healthy individuals. **(D)** Comparison of serum PPIH levels between GC patients and healthy individuals

To ascertain the potential of PPIH protein as a clinical tumor marker, we conducted an enzyme-linked immunosorbent assay (ELISA) to measure PPIH levels in serum samples from patients with LIHC, COAD, BC, and healthy controls, comprising 16 cases each. Additionally, serum from 16 gastric cancer (GC) patients was analyzed. The ELISA results indicated that serum PPIH levels in the LIHC, COAD, BC, GC, and the healthy control group were 230.32 ± 12.57 pg/ml, 220.83 ± 11.90 pg/ml, 225.17 ± 14.97 pg/ml, 230.31 ± 14.36 pg/ml, and 250.01 ± 11.49 pg/ml, respectively. Compared with the healthy control group, serum PPIH levels were significantly down-regulated in patients with LIHC, COAD, BC, and GC (Fig. 7A-D). The differences were statistically significant, with *p*-values indicating increasing levels of significance (*p < 0.05; **p < 0.01; ***p < 0.001; ****p < 0.0001).

#### ROC analysis of PPIH combined with conventional protein markers for cancer diagnosis

**Fig. 8:**
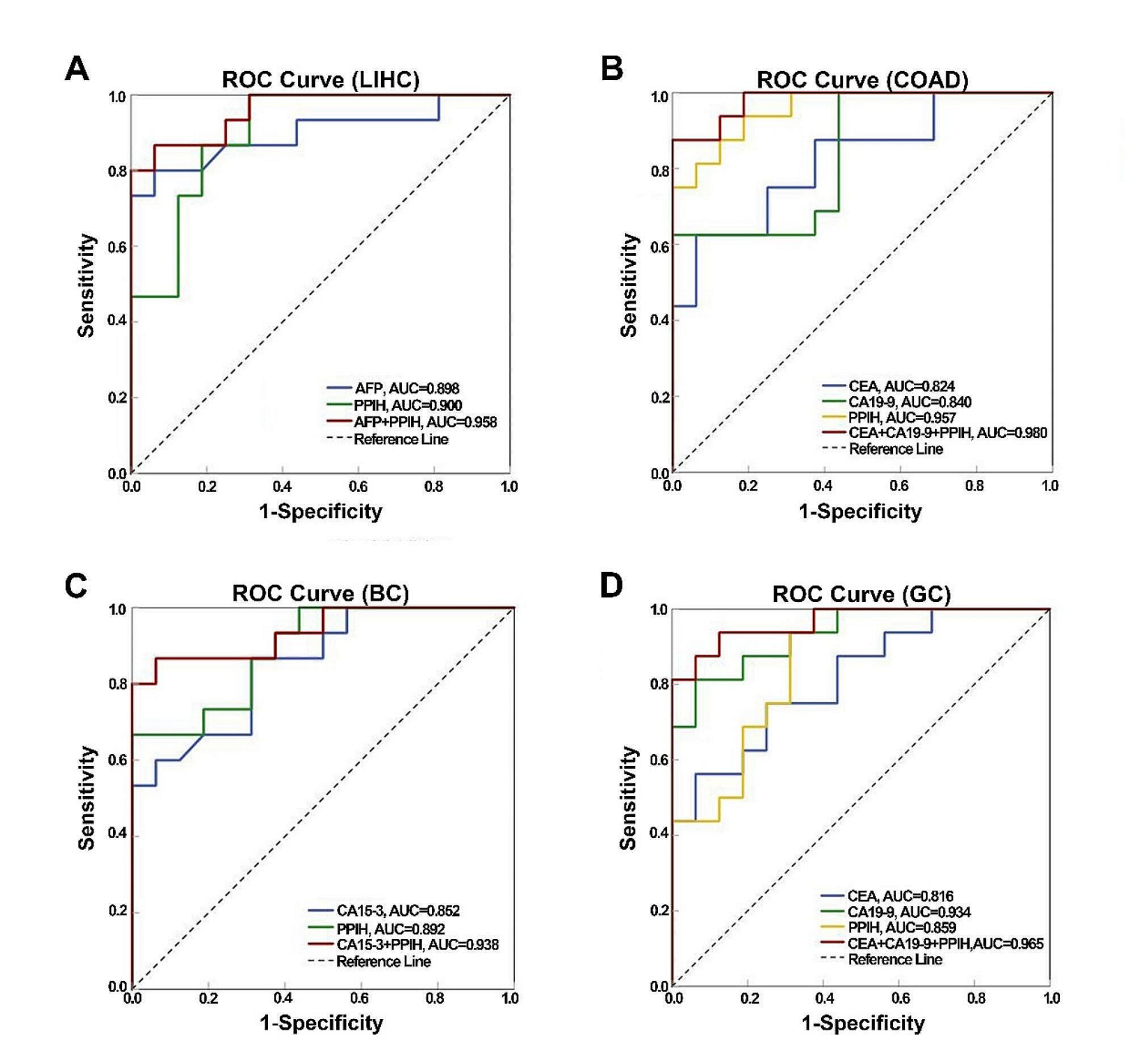
ROC curve analysis for diagnosing four types of cancer using serum PPIH combined with tumor markers. **(A)** ROC curve analysis of serum PPIH combined with AFP for diagnosing LIHC. **(B)** ROC curve analysis of serum PPIH combined with CEA and CA19-9 for diagnosing COAD. **(C)** ROC curve analysis of serum PPIH combined with CA15-3 for diagnosing BC. **(D)** ROC curve analysis of serum PPIH combined with CEA and CA19-9 for diagnosing GC.

We evaluated PPIH’s potential as a serological marker to distinguish cancer patients from healthy individuals. The Receiver Operating Characteristic (ROC) curve analysis of PPIH, combined with traditional tumor markers, was conducted for the diagnosis of four types of cancers. For LIHC diagnosis, AFP showed a sensitivity of 80.0% and a specificity of 93.8%, with an area under the curve (AUC) of 0.898 (95%CI: 0.777, 1.000). PPIH alone had a sensitivity of 100.0% and a specificity of 68.8%, with an AUC of 0.900 (95%CI: 0.793, 1.000). The combination of AFP and PPIH improved the diagnostic performance with a sensitivity of 86.7%, specificity of 93.8%, and an AUC of 0.958 (95%CI: 0.896, 1.000) (Fig. 8A). In COAD diagnosis, CEA had a sensitivity of 62.5% and specificity of 93.8%, with an AUC of 0.824 (95%CI: 0.680, 0.968). CA19-9’s sensitivity was 62.5%, specificity was 100.0%, and AUC was 0.840 (95%CI: 0.701, 0.978). PPIH alone had a sensitivity of 75.0%, specificity of 100.0%, and AUC of 0.957 (95%CI: 0.897, 1.000). Combining CEA, CA19-9, and PPIH resulted in a sensitivity of 87.5%, specificity of 100.0%, and an AUC of 0.980 (95%CI: 0.944, 1.000) (Fig. 8B). For BC, CA15-3 alone offered a sensitivity of 86.7%, specificity of 68.8%, and AUC of 0.852 (95%CI: 0.721, 0.983). PPIH’s sensitivity was 66.7%, specificity was 100.0%, and AUC was 0.892 (95%CI: 0.782, 1.000). The combination of CA15-3 and PPIH yielded a sensitivity of 86.7%, specificity of 93.8%, and an AUC of 0.938 (95%CI: 0.852, 1.000) (Fig. 8C). In GC diagnosis, CEA had a sensitivity of 56.3%, specificity of 93.8%, and an AUC of 0.816 (95%CI: 0.672, 0.961). CA19-9’s sensitivity was 81.3%, specificity was 93.8%, and AUC was 0.934 (95%CI: 0.853, 1.000). PPIH alone showed a sensitivity of 93.8%, specificity of 68.8%, and an AUC of 0.859 (95%CI: 0.732, 0.987). The combined use of CEA, CA19-9, and PPIH provided a sensitivity of 93.8%, specificity of 87.5%, and an AUC of 0.965 (95%CI: 0.909, 1.000) for GC (Fig. 8D). These results demonstrate that PPIH, particularly when used in conjunction with traditional tumor markers, offers better sensitivity and specificity as a serum marker for cancer.

## Discussion

The rapid growth and aging of the global population have positioned cancer as a foremost cause of death and a significant contributor to disability worldwide, with new cases continually escalating and imposing a substantial societal burden [18]. Notably, the incidence and mortality rates of LIHC, COAD, BC, and GC rank highly globally [19–22], with BC emerging as a significant health challenge for women [23]. Currently, tumor clinical management predominantly utilizes a combination of surgery, radiotherapy, chemotherapy, immunotherapy, and targeted drug therapy. These treatments aim to enhance patient survival rates; however, associated cytotoxicity and drug resistance, arising from treatment side effects, often result in low survival rates [24], It is well-established that early tumor diagnosis and subsequent treatment substantially improve patient prognoses [25], underscoring the critical need for novel diagnostic markers and therapeutic target molecules in cancer care.

Although prior research has established *PPIH* ‘s involvement in the progression of certain malignancies [10–12], the specifics of its expression, impact on patient prognosis, and regulatory mechanisms are not fully elucidated. Our study concentrates on *PPIH* ‘s aberrant expression in LIHC, COAD, BC, and GC to discern its correlation with clinicopathological characteristics. We also aim to analyze prognostic factors that influence patient outcomes and the feasibility of developing new tumor targets. By leveraging bioinformatics and validating with clinical specimens, we aimed to clarify *PPIH* ‘s molecular role and its relationship with common solid tumor progression. We first examined mRNA sequencing data from the TCGA database, where we detected significant differences in *PPIH* expression between tumors and normal tissues for 16 cancers (Fig. 1A), after which we focused on evaluating samples from LIHC, COAD, and BC. This study presents novel evidence of significantly elevated *PPIH* mRNA in BC, correlating with poor prognosis (Figs. 1C and 3B). Peng et al. reported a similar association between the upregulation of Cyclophilin A (CypA) and a negative prognosis in colorectal cancer [26], and both *PPIH* and CypA are Cyclophilins. However, our findings showed no significant correlation between *PPIH* mRNA overexpression in COAD and prognosis, likely due to limited sample sizes (Figs. 1B and 3A), prompting the need for larger sample studies in the future. Previous studies have indicated higher *PPIH* mRNA levels in LIHC compared to healthy liver tissue [27, 28], which is consistent with our findings (Fig. 1D). Additionally, high *PPIH* mRNA levels were strongly linked to poor LIHC prognosis [13]. We also validated abnormal PPIH expression in human liver cancer tissues (Fig. 1E-F), and immunohistochemical analyses revealed a correlation between PPIH protein expression and mRNA levels in LIHC, COAD and BC specimens (Fig. 2). These insights propose *PPIH* as a potential biomarker for the early detection and prognostic assessment of common solid tumors.

Pathway analyses of *PPIH*-associated co-expressed genes in BC and LIHC patients implicated *PPIH* in cell cycle control, spliceosome function, and RNA degradation (Figs. 4C-D and 5). These findings align with previous research [8, 13], suggesting that *PPIH* may significantly influence tumor development through the spliceosome pathway. In our previous research, Further investigations into *PPIH*’s interactome, constructed via STRING and GeneMANIA, identified pre-mRNA processing factors (PRPF3, PRPF31, and PRPF4) as intersection points, reinforcing *PPIH*’s pivotal role in tumorigenesis. Additionally, we theorize that substantial downregulation of N6-methyladenosine modification levels in *PPIH* mRNA could be a primary factor in the observed overexpression of *PPIH* in tumors [13].

Our investigation identified critical networks of target miRNAs and transcription factors linked to the differential expression of *PPIH* in liver hepatocellular carcinoma (LIHC), as shown in Fig. 6. We discovered that potential miRNAs regulating *PPIH* expression in LIHC include miR-202, miR-129, miR-141, miR-200a, miR-199a, miR-30a-3p, and miR-30e-3p. Zhuang et al. [29] reported that miR-202 acts as a tumor suppressor in hepatocellular carcinoma by reducing BCL2 expression, noting a significant decrease in miR-202 in hepatocellular tissues and cell lines, with a concomitant increase in BCL2 expression. Zhai et al. [30] found that miR-129 hampers tumor initiation and progression by targeting PAK5, whereas Zhao et al. [31] observed that miR-141 curbs hepatocellular carcinoma cell proliferation and invasion by downregulating TGFβR1. According to Liu et al. [32], Circ-ZEB1 and PIK3CA are markers of poor prognosis in hepatocellular carcinoma, with low levels of MiR-199a-3p correlating with reduced tumor cell proliferation and increased apoptosis. Wang et al. [33] demonstrated the tumor-suppressive role of miR-30a-3p in LIHC. Our research suggests GGAANCGGAANY_UNKNOWN, V$PAX4_02, and SCGGAAGY_V$ELK1_02 as key transcription factors in *PPIH* regulation. Ma et al. [34] described how the transcription co-activator P300 promotes malignancy in hepatocellular carcinoma by enhancing αPKC-ι expression, mediated by Elk1. The novel transcription factor target (GGAANCGGAANY_UNKNOWN) identified in our study has not been previously reported. Collectively, our data suggest that aberrant *PPIH* expression may influence tumor cell proliferation, invasion, and metastasis by modulating these targets, warranting further verification.

Serological markers offer significant benefits for tumor diagnosis and monitoring, such as non-invasiveness and reproducibility. Tumor detection via serum proteomics provides a sensitive and informative alternative to tissue samples due to its systemic perspective [35]. Alpha-fetoprotein (AFP) is a specific serum glycoprotein vital for liver cancer diagnosis; however, its sensitivity and specificity are limited. Its detection alongside liver ultrasound can facilitate early liver cancer detection in high-risk groups [36–38]. CEA and CA19-9, while commonly used in clinical settings to predict GC outcomes, have unsatisfactory positive rates [38–40]. Similarly, CA15-3, despite being a classical BC marker, is not always indicative of early-stage BC and may elevate in benign conditions [41]. Thus, identifying more precise tumor markers remains crucial. This study marks the first evaluation of PPIH levels in the serum of patients with LIHC, COAD, BC and GC, revealing lower PPIH levels in patients compared to healthy controls, a finding that contrasts with earlier tissue-based results (Fig. 7).

Circular RNA (circRNA) is a member of non-coding RNAs, and currently, circRNA is thought to be generated by selective reverse splicing of pre- mRNA [42]. CircRNA is implicated in various tumorigenic processes and acts as an epigenetic regulator [43]. Deng et al. [44] observed that CircSKA3 is abundant in colorectal cancer tissues but reduced in serum, potentially due to transcription factor SLUG interactions that affect circSKA3’s cellular retention and secretion. The biological functions of circRNA include regulating gene transcription or RNA splicing in the nucleus, binding with RNA binding protein (RBP), etc [45]. The interaction between circRNA and protein will affect the synthesis and degradation of circRNA and the expression and function of protein [46]. Our analysis of *PPIH* co-expressed genes suggests a significant role in RNA binding, positing that elevated PPIH in tumor tissues and reduced serum levels might be attributable to PPIH’s interaction with circRNA, fostering tumor cell advantages while decreasing PPIH secretion. This theory requires further investigation.

In our study, combining traditional tumor markers (AFP, CEA, CA19-9, CA15-3) with PPIH improved diagnostic accuracy for LIHC, COAD, BC, and GC, as depicted in Fig. 8. Nevertheless, a lack of significant correlation between PPIH expression and clinicopathological features due to limited patient data points to the need for broader clinical validation (Supplementary Tables 7–10).

In summary, we found that *PPIH* is upregulated in common solid tumors and correlates with poor prognosis, possibly facilitating tumor development via the spliceosome pathway. Despite reduced serum PPIH levels in patients with tumors, suggesting its potential as a biomarker, the inverse expression pattern of *PPIH* in tissues and serum merits further study to substantiate our conjectures.

### Electronic supplementary material

Below is the link to the electronic supplementary material.

Supplementary Material 1

Supplementary Material 2

## Data Availability

All data generated or analysed during this study are included in this published article [and its supplementary information files].
