# Supplementary material for: PPIH acts as a potential predictive biomarker for patients with common solid tumors
Source: BMC Cancer. 2024 Jun 4;24:681. doi: 10.1186/s12885-024-12446-9 (PMC11151604; doi:10.1186/s12885-024-12446-9)
Supplement: Supplementary file 1 — Supplementary Material 1 [file 12885_2024_12446_MOESM1_ESM.docx]

**Supplementary Table 1. Significantly enriched GO annotations (biological processes) related to PPIH in LIHC (LinkedOmics).**

| **Description** | **Leading Edge**  **Number** | **FDR** | **Leading Edge Gene** |
| --- | --- | --- | --- |
| ribonucleoprotein complex biogenesis | 167 | 0 | AATF; BRIX1; BYSL; CD2BP2; DCAF13; DDX27; DDX49; DDX51; DDX56; DENR; EBNA1BP2; EIF3B; EIF3CL; EIF3D; EIF3F; EIF3G; EIF3H; EIF3I; EIF3K; EIF3M; EIF6; EMG1; ERAL1; ERI3; EXOSC1; EXOSC10; EXOSC2; EXOSC3; EXOSC4; EXOSC5; EXOSC6; EXOSC7; EXOSC8; EXOSC9; FBL; FRG1; FTSJ3; GAR1; GEMIN6; GEMIN7; GNL2; GTF2H5; GTF3A; IMP3; IMP4; KRI1; LAS1L; LSM3; LSM4; LSM6; LUC7L; LYAR; MCTS1; MPHOSPH6; MRPL11; MRPL20; MRPL22; MRPS11; MRPS2; MRPS7; MRPS9; MRTO4; NHP2; NLE1; NOB1; NOC2L; NOC4L; NOL10; NOL12; NOP10; NOP16; NOP2; NOP56; NOP58; NPM1; NSUN4; NSUN5; PA2G4; PELP1; PES1; PIH1D1; PIH1D2; POLR2D; POP4; POP5; PPAN; PRMT7; PRPF19; PRPF31; PRPF6; PWP1; RAN; RBM22; RBMX; REXO4; RPF1; RPF2; RPL10; RPL10A; RPL11; RPL12; RPL13A; RPL14; RPL23A; RPL24; RPL26; RPL26L1; RPL27; RPL35; RPL35A; RPL38; RPL5; RPL6; RPL7A; RPLP0; RPS10; RPS14; RPS15; RPS16; RPS17; RPS19; RPS2; RPS21; RPS23; RPS24; RPS5; RPS6; RPS7; RPS8; RPS9; RPSA; RRP1; RRP7A; RRP8; RRP9; RSL1D1; RSL24D1; RUVBL1; RUVBL2; SART1; SF3A2; SF3A3; SNRPB; SNRPC; SNRPD1; SNRPD2; SNRPD3; SNRPE; SNRPF; SNRPG; STYXL1; SURF6; SUV39H1; TARBP2; TBL3; TRMT112; TXNL4A; UTP14A; UTP6; VCX; WDR46; WDR74; WDR75; ZNF593; ZNF622; ZNHIT3; ZRSR2 |
| mitochondrial gene expression | 66 | 0 | AURKAIP1; CDK5RAP1; CHCHD1; ERAL1; GADD45GIP1; GARS; HARS; MRPL1; MRPL10; MRPL11; MRPL12; MRPL13; MRPL14; MRPL17; MRPL18; MRPL2; MRPL20; MRPL21; MRPL22; MRPL23; MRPL24; MRPL27; MRPL28; MRPL33; MRPL36; MRPL37; MRPL38; MRPL41; MRPL43; MRPL47; MRPL48; MRPL50; MRPL51; MRPL52; MRPL53; MRPL55; MRPL9; MRPS11; MRPS12; MRPS15; MRPS16; MRPS17; MRPS18A; MRPS18C; MRPS2; MRPS21; MRPS23; MRPS24; MRPS25; MRPS26; MRPS30; MRPS33; MRPS34; MRPS5; MRPS7; MRPS9; NDUFA7; NSUN4; PUS1; RPUSD3; SUPV3L1; TBRG4; TRIT1; TRUB2; TUFM; WARS2 |
| mRNA processing | 126 | 0 | AKAP8L; ARL6IP4; AURKAIP1; BUD31; CCNB1; CD2BP2; CDK9; CPSF3; CPSF4; CWC15; CWC27; DAZAP1; DDX41; DNAJC8; EFTUD2; FRG1; FUS; GEMIN6; GEMIN7; GEMIN8; GTF2H5; H2AFB1; HMX2; HNRNPA1; HNRNPA3; HNRNPC; HNRNPD; HNRNPL; HNRNPM; HSF1; IK; JMJD6; KDM1A; KHDRBS1; LSM10; LSM2; LSM3; LSM4; LSM5; LSM6; LSM7; LUC7L; MAGOH; MAGOHB; NCBP2; PABPN1; PAF1; POLR2D; POLR2F; POLR2G; POLR2H; POLR2I; POLR2J; POLR2K; POLR2L; PPIE; PPIH; PPIL1; PQBP1; PRCC; PRMT7; PRPF19; PRPF3; PRPF31; PRPF38A; PRPF4; PRPF6; PUF60; RALY; RBM10; RBM15; RBM17; RBM22; RBM3; RBM38; RBM4; RBM42; RBMX; RBMX2; RNF113A; RNPS1; SARNP; SART1; SF3A2; SF3A3; SF3B4; SF3B5; SFPQ; SNRNP25; SNRNP35; SNRNP40; SNRNP70; SNRPA; SNRPA1; SNRPB; SNRPB2; SNRPC; SNRPD1; SNRPD2; SNRPD3; SNRPE; SNRPF; SNRPG; SRRT; SSU72; SUPT5H; SYF2; SYMPK; TARDBP; TBRG4; THOC1; THOC3; THOC5; THOC6; THOC7; TSEN34; TSEN54; TXNL4A; U2AF1L4; U2AF2; UBL5; WDR83; XAB2; YBX1; ZMAT5; ZRSR2 |
| protein targeting | 113 | 0 | ACOT8; AP4B1; AP4M1; CHMP4B; CIB1; DNLZ; GABARAP; HRAS; HTRA2; IMMP1L; ITGB1BP1; MPV17; NDUFA13; PARL; PDCD5; PMM2; ROMO1; RPL10; RPL10A; RPL11; RPL12; RPL13; RPL13A; RPL14; RPL15; RPL17; RPL18; RPL18A; RPL19; RPL21; RPL22; RPL23; RPL23A; RPL24; RPL26; RPL27; RPL27A; RPL28; RPL29; RPL30; RPL31; RPL32; RPL34; RPL35; RPL35A; RPL36; RPL37; RPL37A; RPL38; RPL39; RPL4; RPL41; RPL5; RPL6; RPL7A; RPL8; RPLP0; RPLP1; RPLP2; RPS10; RPS11; RPS12; RPS13; RPS14; RPS15; RPS15A; RPS16; RPS17; RPS18; RPS19; RPS2; RPS20; RPS21; RPS23; RPS24; RPS25; RPS26; RPS27A; RPS29; RPS3; RPS3A; RPS4X; RPS5; RPS6; RPS7; RPS8; RPS9; RPSA; SAE1; SEC61B; SEC61G; SLC25A6; SRP14; SRP19; SSR2; TIMM10; TIMM17B; TIMM22; TIMM50; TIMM8B; TIMM9; TOMM22; TOMM40; TOMM5; TOMM7; UBA52; UBE2D2; UBE2J2; UBE2L3; UBL5; VPS4A; ZDHHC12; ZFAND2B |
| tRNA metabolic process | 63 | 0.00030353 | ADAT3; AIMP1; CDK5RAP1; CTU1; CTU2; DALRD3; DTD1; EEF1E1; EXOSC2; EXOSC3; EXOSC7; EXOSC8; EXOSC9; FARSA; FARSB; FTSJ1; GARS; GTF3C5; GTF3C6; HARS; KARS; LAGE3; LSM6; MARS; METTL1; METTL6; OSGEP; PARS2; POLR3K; POP1; POP4; POP5; POP7; PUS1; PUSL1; QARS; RARS; RPP21; RPP25; RPP30; RPP38; SARS; SARS2; SLFN13; SSB; THADA; THUMPD2; TP53RK; TPRKB; TRIT1; TRMT1; TRMT112; TRMT12; TRMT61A; TRMU; TRPT1; TSEN34; TSEN54; URM1; WARS2; WDR4; YARS; ZBTB8OS |

Abbreviations: FDR, false discovery rate from Benjamini and Hochberg from gene set enrichment analysis (GSEA).

**Supplementary Table 2. Significantly enriched GO annotations (cellular components) related to PPIH in LIHC (LinkedOmics).**

| **Description** | **Leading Edge**  **Number** | **FDR** | **Leading Edge Gene** |
| --- | --- | --- | --- |
| spliceosomal complex | 55 | 0 | BUD31; CWC15; CWC27; DDX41; EFTUD2; FRG1; HNRNPA1; HNRNPA3; HNRNPC; IK; LSM2; LSM3; LSM4; LSM5; LSM6; LSM7; LUC7L; MAGOH; MAGOHB; PPIE; PPIH; PRPF19; PRPF31; PRPF38A; PRPF6; RALY; RBM17; RBM22; RBMX; RNF113A; SART1; SF3A2; SF3A3; SF3B5; SNRNP25; SNRNP35; SNRNP40; SNRNP70; SNRPA; SNRPA1; SNRPB; SNRPB2; SNRPC; SNRPD1; SNRPD2; SNRPD3; SNRPE; SNRPF; SNRPG; SYF2; TXNL4A; U2AF1L4; YBX1; ZMAT5; ZRSR2 |
| cytosolic part | 107 | 0 | BCAS4; BLOC1S1; BLOC1S3; CCT2; CCT3; CCT4; CCT5; CCT6A; CCT7; CTU1; DBN1; DTNBP1; ENO1; ENO2; GET4; GSDMD; HBE1; MCTS1; MRPL1; NAA10; NAA11; PIN1; PRKCG; PSMC4; PSMC5; PSMD14; PYCARD; RPL10; RPL10A; RPL11; RPL12; RPL13; RPL13A; RPL14; RPL15; RPL17; RPL18; RPL18A; RPL19; RPL21; RPL22; RPL22L1; RPL23; RPL23A; RPL24; RPL26; RPL26L1; RPL27; RPL27A; RPL28; RPL29; RPL30; RPL31; RPL32; RPL34; RPL35; RPL35A; RPL36; RPL36A; RPL36AL; RPL37; RPL37A; RPL38; RPL39; RPL4; RPL41; RPL5; RPL6; RPL7A; RPL8; RPLP0; RPLP1; RPLP2; RPS10; RPS11; RPS12; RPS13; RPS14; RPS15; RPS15A; RPS16; RPS17; RPS18; RPS19; RPS2; RPS20; RPS21; RPS23; RPS24; RPS25; RPS26; RPS27A; RPS29; RPS3; RPS3A; RPS4X; RPS5; RPS6; RPS7; RPS8; RPS9; RPSA; RSL24D1; SURF6; TCP1; UBA52; ZNF622 |
| mitochondrial inner membrane | 141 | 0 | ATAD3A; ATAD3B; AURKAIP1; CHCHD1; CHCHD6; COQ3; COQ4; COX16; COX4I1; COX5A; COX5B; COX6A1; COX6B1; COX6C; COX7A2; COX7B; COX7C; COX8A; CYC1; ERAL1; FPGS; GRPEL2; HIGD2A; IMMP1L; MPV17; MRPL1; MRPL10; MRPL11; MRPL12; MRPL13; MRPL14; MRPL17; MRPL18; MRPL2; MRPL20; MRPL21; MRPL22; MRPL23; MRPL24; MRPL27; MRPL28; MRPL33; MRPL36; MRPL37; MRPL38; MRPL41; MRPL43; MRPL47; MRPL48; MRPL50; MRPL51; MRPL52; MRPL53; MRPL55; MRPL9; MRPS11; MRPS12; MRPS15; MRPS16; MRPS17; MRPS18A; MRPS18C; MRPS2; MRPS21; MRPS23; MRPS24; MRPS25; MRPS26; MRPS30; MRPS33; MRPS34; MRPS5; MRPS7; MRPS9; NDUFA1; NDUFA11; NDUFA12; NDUFA13; NDUFA2; NDUFA3; NDUFA4L2; NDUFA7; NDUFA8; NDUFA9; NDUFAB1; NDUFAF2; NDUFAF3; NDUFAF4; NDUFB1; NDUFB10; NDUFB11; NDUFB2; NDUFB3; NDUFB4; NDUFB5; NDUFB6; NDUFB7; NDUFB9; NDUFC1; NDUFS3; NDUFS5; NDUFS6; NDUFS8; PARK7; PARL; PHB; PMPCA; PPOX; PTPMT1; ROMO1; RPS3; SCO2; SLC25A14; SLC25A19; SLC25A3; SLC25A39; SLC25A6; SPNS1; STOML2; SURF1; TAZ; TIMM10; TIMM13; TIMM17B; TIMM22; TIMM50; TIMM8B; TIMM9; TMEM11; TMEM126A; TMEM14C; TOMM40; TYMS; UCP2; UQCR10; UQCR11; UQCRB; UQCRH; UQCRHL; UQCRQ; WDR93 |
| chromosomal region | 109 | 0.00023915 | ACD; AURKB; AURKC; BIRC5; BLM; BOD1; BUB1; BUB1B; CBX3; CCNB1; CDCA5; CDCA8; CDK1; CDT1; CENPA; CENPB; CENPH; CENPM; CENPN; CENPP; CENPT; CENPW; CFDP1; CHEK1; CHEK2; DAXX; DCLRE1C; DCTN2; DCTN3; DCTN5; DNMT3A; DSCC1; DSN1; DYNC1LI1; DYNLL1; ERCC1; ERCC6L; FEN1; GAR1; H2AFX; H2AFY; H3F3A; HELLS; HJURP; HNRNPA2B1; HNRNPU; HSF1; ITGB3BP; KDM1A; KIF18A; KIF22; KIF2C; MAD1L1; MAD2L1; MCM2; MCM3; MCM5; MCM6; MCM7; NCAPD2; NCAPG; NDC80; NEK2; NHP2; NLRP2; NSMCE1; NSMCE2; NUF2; NUP37; NUP43; NUP85; OIP5; PCNA; PIF1; PLK1; PML; POLD1; PPP1CA; PPP1CC; PPP2R1A; PTGES3; RAD51; RANGAP1; RCC2; REC8; RECQL4; RPA2; SCMH1; SEC13; SIRT6; SKA1; SKA3; SPC25; SSB; SUGT1; SUV39H1; TELO2; TERT; THOC1; THOC3; THOC5; THOC6; THOC7; TTK; WRAP53; XRCC1; XRCC6; ZBTB48; ZWINT |

**Supplementary Table 3. Significantly enriched GO annotations (molecular functions) related to PPIH in LIHC (LinkedOmics).**

| **Description** | **Leading Edge**  **Number** | **FDR** | **Leading Edge Gene** |
| --- | --- | --- | --- |
| structural constituent of ribosome | 111 | 0 | MRPL11; MRPL12; MRPL13; MRPL14; MRPL17; MRPL18; MRPL20; MRPL21; MRPL22; MRPL23; MRPL27; MRPL28; MRPL33; MRPL36; MRPL37; MRPL41; MRPL47; MRPL51; MRPL52; MRPL55; MRPL9; MRPS11; MRPS12; MRPS15; MRPS16; MRPS17; MRPS18A; MRPS2; MRPS21; MRPS23; MRPS24; MRPS30; MRPS34; MRPS5; MRPS7; MRPS9; NDUFA7; RPL10; RPL10A; RPL11; RPL12; RPL13; RPL13A; RPL14; RPL15; RPL17; RPL18; RPL18A; RPL19; RPL21; RPL22; RPL22L1; RPL23; RPL23A; RPL24; RPL26; RPL26L1; RPL27; RPL27A; RPL28; RPL29; RPL30; RPL31; RPL32; RPL34; RPL35; RPL35A; RPL36; RPL37; RPL37A; RPL38; RPL39; RPL4; RPL41; RPL5; RPL6; RPL7A; RPL8; RPLP0; RPLP1; RPLP2; RPS10; RPS11; RPS12; RPS13; RPS14; RPS15; RPS15A; RPS16; RPS17; RPS18; RPS19; RPS2; RPS20; RPS21; RPS23; RPS24; RPS26; RPS27A; RPS29; RPS3; RPS3A; RPS4X; RPS5; RPS6; RPS7; RPS8; RPS9; RPSA; RSL24D1; UBA52 |
| unfolded protein binding | 37 | 0.0023995 | AIP; CCT2; CCT3; CCT4; CCT5; CCT6A; CCT7; CDC37; CHAF1A; CHAF1B; DNAJB13; DNAJB2; GRPEL2; HSPA6; HSPE1; HTRA2; MKKS; NAP1L4; NPM1; NUDC; PDRG1; PFDN1; PFDN2; PFDN4; PFDN5; PFDN6; PPIA; PPIAL4C; PPIAL4G; PPIB; PPIE; PPIH; PTGES3; RUVBL2; SERPINH1; TCP1; TTC1 |
| catalytic activity, acting on RNA | 98 | 0.011464 | CPSF3; DALRD3; DDX54; DDX56; DHX34; DTD1; EDC3; EIF4A1; EIF4A3; EMG1; ERI3; EXO1; EXOSC1; EXOSC10; EXOSC2; EXOSC3; EXOSC4; EXOSC5; EXOSC6; EXOSC7; EXOSC8; EXOSC9; FARSA; FARSB; FBL; FEN1; FTSJ1; FTSJ3; GARS; HARS; ISG20; KARS; MARS; METTL1; METTL6; MOV10; NOB1; NOP2; NSUN4; PARS2; PIF1; POLR1C; POLR1D; POLR2D; POLR2F; POLR2G; POLR2H; POLR2I; POLR2J; POLR2K; POLR2L; POLR3C; POLR3F; POLR3H; POLR3K; POP1; POP4; POP5; POP7; PTRH1; PTRH2; PUS1; QARS; QTRT1; RAD54B; RARS; RBMX2; RNASE1; RNASE6; RNASEH1; RNASEH2A; RNASEH2B; RNASEK; RNASET2; RPP21; RPP25; RPP30; RPP38; SARS; SARS2; SLFN13; SUPV3L1; TERT; THUMPD2; TOE1; TRIT1; TRMT1; TRMT112; TRMT12; TRMT61A; TRPT1; TSEN34; TSEN54; WARS; WARS2; WDR4; YARS; ZNRD1 |

**Supplementary Table 4. Significantly enriched KEGG pathway annotations related to PPIH in LIHC (LinkedOmics).**

| **Description** | **Leading Edge**  **Number** | **FDR** | **Leading Edge Gene** |
| --- | --- | --- | --- |
| Ribosome | 100 | 0 | FAU; MRPL11; MRPL12; MRPL13; MRPL14; MRPL17; MRPL18; MRPL20; MRPL21; MRPL22; MRPL23; MRPL27; MRPL28; MRPL33; MRPL36; MRPL9; MRPS12; MRPS15; MRPS16; MRPS17; MRPS18A; MRPS2; MRPS21; MRPS5; MRPS7; MRPS9; RPL10; RPL10A; RPL11; RPL12; RPL13; RPL13A; RPL14; RPL15; RPL17; RPL18; RPL18A; RPL19; RPL21; RPL22; RPL23; RPL23A; RPL24; RPL26; RPL26L1; RPL27; RPL27A; RPL28; RPL29; RPL30; RPL31; RPL32; RPL34; RPL35; RPL35A; RPL36; RPL37; RPL37A; RPL38; RPL39; RPL4; RPL41; RPL5; RPL6; RPL7A; RPL8; RPLP0; RPLP1; RPLP2; RPS10; RPS11; RPS12; RPS13; RPS14; RPS15; RPS15A; RPS16; RPS17; RPS18; RPS19; RPS2; RPS20; RPS21; RPS23; RPS24; RPS25; RPS26; RPS27A; RPS29; RPS3; RPS3A; RPS4X; RPS5; RPS6; RPS7; RPS8; RPS9; RPSA; RSL24D1; UBA52 |
| Spliceosome | 61 | 0 | BUD31; CCDC12; CWC15; EFTUD2; HNRNPA1; HNRNPA3; HNRNPC; HNRNPM; HSPA1B; HSPA6; ISY1; LSM2; LSM3; LSM4; LSM5; LSM6; LSM7; MAGOH; MAGOHB; NCBP2; PPIE; PPIH; PPIL1; PQBP1; PRPF19; PRPF3; PRPF31; PRPF38A; PRPF4; PRPF6; PUF60; RBM17; RBM22; RBMX; RP9; SART1; SF3A2; SF3A3; SF3B4; SF3B5; SNRNP40; SNRNP70; SNRPA; SNRPA1; SNRPB; SNRPB2; SNRPC; SNRPD1; SNRPD2; SNRPD3; SNRPE; SNRPF; SNRPG; SYF2; THOC1; THOC3; TXNL4A; U2AF1; U2AF1L4; U2AF2; XAB2 |
| Huntington disease | 61 | 0 | AP2M1; AP2S1; BAX; CLTA; CLTB; COX4I1; COX5A; COX5B; COX6A1; COX6B1; COX6C; COX7A2; COX7B; COX7C; COX8A; CREB3; CYC1; DCTN2; HDAC1; HDAC2; NDUFA1; NDUFA11; NDUFA12; NDUFA13; NDUFA2; NDUFA3; NDUFA4L2; NDUFA7; NDUFA8; NDUFA9; NDUFAB1; NDUFB1; NDUFB10; NDUFB11; NDUFB2; NDUFB3; NDUFB4; NDUFB5; NDUFB6; NDUFB7; NDUFB9; NDUFC1; NDUFS3; NDUFS5; NDUFS6; NDUFS8; NRF1; POLR2D; POLR2F; POLR2G; POLR2H; POLR2I; POLR2J; POLR2K; POLR2L; SLC25A6; UQCR10; UQCRB; UQCRH; UQCRHL; VDAC2 |
| RNA transport | 53 | 0.00039300 | AAAS; EEF1A1; EIF1; EIF2B1; EIF2B3; EIF2B4; EIF2B5; EIF2S2; EIF3B; EIF3CL; EIF3D; EIF3F; EIF3G; EIF3H; EIF3I; EIF4A1; EIF4E2; EIF4EBP1; EIF5B; GEMIN6; GEMIN7; GEMIN8; MAGOH; MAGOHB; NCBP2; NUP37; NUP43; NUP62; NUP85; NUP93; NXT1; PABPC4; POP1; POP4; POP5; POP7; RAE1; RAN; RNPS1; RPP21; RPP25; RPP30; RPP38; SEC13; SUMO1; SUMO2; TACC3; THOC1; THOC3; THOC5; THOC6; THOC7; UBE2I |
| Cell cycle | 51 | 0.0046506 | ANAPC11; ANAPC5; ANAPC7; BUB1; BUB1B; CCNA2; CCNB1; CCNB2; CCND3; CCNE1; CDC20; CDC25A; CDC25B; CDC25C; CDC45; CDC6; CDC7; CDK1; CDK4; CDK7; CDKN2A; CDKN2C; CDKN2D; CHEK1; CHEK2; DBF4; E2F1; E2F2; E2F4; HDAC1; HDAC2; MAD1L1; MAD2L1; MAD2L2; MCM2; MCM3; MCM5; MCM6; MCM7; PCNA; PKMYT1; PLK1; PTTG1; PTTG2; RBX1; SFN; TFDP2; TTK; YWHAE; YWHAZ; ZBTB17 |
| Systemic lupus erythematosus | 52 | 0.010956 | C1QA; C1QB; C1QC; CD80; CD86; FCGR1A; FCGR2A; FCGR3A; H2AFB1; H2AFJ; H2AFX; H2AFY; H2AFY2; H2AFZ; H2BFM; H2BFWT; H3F3A; H3F3B; HIST1H2AB; HIST1H2AE; HIST1H2AH; HIST1H2AJ; HIST1H2AL; HIST1H2AM; HIST1H2BG; HIST1H3B; HIST1H3C; HIST1H3D; HIST1H3I; HIST1H4A; HIST1H4B; HIST1H4C; HIST1H4D; HIST1H4E; HIST1H4H; HIST2H2AA3; HIST2H2AB; HIST2H2AC; HIST2H3C; HIST2H4A; HLA-DMA; HLA-DMB; HLA-DOB; HLA-DPB1; HLA-DQA2; HLA-DQB1; HLA-DRB1; IFNG; SNRPB; SNRPD1; SNRPD3; SSB |
| Purine metabolism | 46 | 0.013806 | ADA; ADSL; AK1; AK2; APRT; ATIC; DGUOK; FHIT; GUK1; HDDC3; IMPDH1; IMPDH2; ITPA; NME1; NME1-NME2; NME2; NME3; NME4; NME6; NME7; NT5C; NUDT2; PDE6B; POLA2; POLD1; POLD2; POLE3; POLE4; POLR1C; POLR1D; POLR1E; POLR2D; POLR2F; POLR2G; POLR2H; POLR2I; POLR2J; POLR2K; POLR2L; POLR3C; POLR3D; POLR3F; POLR3H; POLR3K; RRM2; ZNRD1 |

**Supplementary Table 5. Significantly enriched miRNA-target networks associated with PPIH in LIHC (LinkedOmics).**

| **Geneset** | **Leading Edge Gene** |
| --- | --- |
| ATACCTC,MIR-202 | ABHD13; ADCY9; ADIPOR2; AKAP13; ARL5A; ATG16L1; BCL7A; CALD1; CGN; CHN2; COL15A1; CPEB2; CPEB3; CPEB4; CTDSPL2; CYTH3; DCUN1D4; DICER1; DLC1; DNAJB9; DST; DUSP1; EPHA3; FOXN3; GABPA; GALNT1; GALNT2; GHR; HECTD2; HMGCS1; HOMER2; INO80D; KIF1B; LAMP2; LIMD1; LPGAT1; MED13L; MTMR12; MYRIP; NEK7; NID2; PGAP1; PPARGC1A; RAB11FIP2; RB1; RHOT1; RNF13; RNF165; RORC; SCYL3; SENP2; SERTAD2; SHANK2; SLC30A4; SLC37A4; STARD13; TAB2; TECPR2; TNRC6B; XRN1; YPEL2; ZBTB10; ZC3H11A |
| GCAAAAA,MIR-129 | APC; ARL5A; ARNT; BMPR2; BPTF; CBX7; CEP192; CTNND1; DDX3X; DDX6; DLGAP2; DNAJC13; DUSP10; EFNB2; EFR3A; EP300; ETS1; FAM13B; FAM160B1; FMR1; FNDC5; GALNT1; GLYR1; HERC4; ING3; ITGA6; KDM1B; KDM2A; MAN1A1; MARCH5; MED13L; NIPBL; NR2C2; NR3C2; OXR1; PDLIM5; PDS5A; PDZRN4; PHF12; PIK3R1; PPP1R12A; PSD3; PTP4A1; RAB21; RNF165; RSBN1; RUNX1T1; SCN8A; SGMS1; SLMAP; SLTM; SP3; SP4; STAT5B; SUN2; TAOK3; TMTC4; TRIO; TSHZ1; USP6; ZBTB10; ZFP36L1; ZFX; ZMYM5; ZNF281; ZNF609 |
| CAGTGTT,MIR-141,MIR-200A | ANKFY1; APBB2; ARNTL; ASTN1; ATP2A2; ATP6V1A; ATXN1; ATXN7; BACE1; BAHD1; BHLHE40; C2orf42; CDC42BPB; CDK13; CDK17; CEP120; CHD9; CLASP2; CSNK1G3; DIP2B; DIXDC1; DLC1; DMXL1; DNAJC13; DUSP3; ERRFI1; EXOC5; FAM168B; FAM84B; FOXA1; FOXN3; HIPK1; HLF; HMG20A; HNRNPF; IKZF5; INO80D; IRS2; ITSN1; KALRN; KPNA3; LRRC8A; LYPLA1; MAP2K4; MED13L; MIB1; MTSS1; MYH10; MYRIP; NAMPT; NR2C2; OSBPL11; PAFAH1B2; PDS5B; PDZD2; PEX5; PHLPP1; PHLPP2; PPP1R15B; PSEN1; PTP4A1; PTPRG; RAD23B; RAP2C; RBM7; RMND5A; RNFT1; RTF1; SEPT7; SIK1; SIRT1; SLC1A1; SLC23A2; SLC39A9; SON; SOX5; SP4; SPAG9; STAT5B; STRN; TCF12; THRB; TMEM135; TNKS2; TNRC6B; TNS3; TOP1; TTBK2; UBE3A; USP9X; VPS26B; WDFY3; WDR26; WDR44; YTHDF3; ZCCHC24; ZFR; ZNF609 |
| CTACTGT,MIR-199A | ABCA1; ADAMTSL3; AFF4; AP1G1; APLP2; ARHGAP20; ARHGAP21; ARHGAP24; ARID4B; ARRDC3; ATP1B2; BAZ2B; BTBD7; CALCRL; CCNT2; CDK17; CPEB4; CTDSPL2; CXXC5; DPP4; EDEM3; FAM193A; FN1; GIGYF2; GOLGA4; GPM6A; HECTD2; KCNK1; KDM6A; LIFR; MAN2A1; MAP2K1; MEF2D; MET; MTOR; NFE2L2; NID2; PDS5B; PHF12; PHLPP2; PI4K2B; PPP1R12A; PPP2R5E; QKI; RAB2B; RBL2; RBM25; RNF111; RNF141; RREB1; SECISBP2L; SIK2; SLC4A4; SMARCA2; SPAST; TAB2; TACC2; TMEM161B; TNRC6B; TOP1; ZEB1; ZHX1; ZNF654 |
| ACTGAAA,MIR-30A-3P,MIR-30E-3P | AHCYL2; AKAP9; ANTXR2; AP1G1; AP4E1; APC; ARF6; ARID4A; ARPP19; ATXN1; BAAT; BIRC6; C5orf24; CDC37L1; CLCN5; CREBBP; CSNK1G3; CYTH1; DDX3X; DLG2; DLST; DNAJB14; DNAJC27; ELK3; EP300; EPAS1; EYA3; FAM13B; FCHO2; FGF7; FNDC5; GALNT1; HERC4; HIRA; HNRNPH2; KIAA0232; KRAS; LRRTM2; MAPK6; MEF2D; MLLT10; NR2C2; NR3C1; OSBPL11; PAPOLG; PBRM1; PPP2R5E; PRKAA1; PTEN; PURA; RNF141; RUNX1T1; SP3; SUN2; TMEM47; TNPO3; TNRC6B; TOB1; TOP1; TSC22D2; TWF1; UBE2G1; USP6; VCPIP1; ZFAND5; ZFX; ZFYVE9 |

**Supplementary Table 6. Significantly enriched transcription factor-target networks associated with PPIH in LIHC (LinkedOmics).**

| **Geneset** | **Leading Edge Gene** |
| --- | --- |
| GGAANCGGAANY_UNKNOWN | BANF1; BMS1; CHMP2A; COMMD6; COX6B1; COX7A2; CSNK2B; DLX4; E2F4; EBNA1BP2; EIF1AD; EIF3H; EIF3K; GYS1; MED8; MRPL21; MRPL43; MRPS18A; MRPS21; MRPS23; MTA2; NCBP2; PDAP1; POMP; PRPF3; PSMB4; PTPRCAP; RARS; RNF25; RPL28; RPL38; RRAS; RUVBL2; SDF2; SEC61G; SMUG1; SNRPE; TAF10; U2AF2; UBA52; UBL5; UBXN1; VPS16 |
| V$PAX4_02 | AP1G1; ARL5B; ATM; ATP2A2; AUTS2; C5; CAST; CGN; CHD2; CPEB4; CTAGE1; CXXC5; DIXDC1; EBF2; ELK3; GGCX; HERPUD2; IKZF3; KLF13; LCOR; LDB2; LRP5; MAN1C1; MBNL2; MBNL3; MIER3; MLLT10; MTMR12; MTMR6; NAALADL2; NFIX; NNAT; NPAT; PITPNM2; PTPN21; PTPRG; RAB6A; RALGPS2; RAPH1; RNF128; RORA; SLC20A2; SMOC1; SOX5; TBC1D8B; TECTA; TOB1; USP32; ZMIZ1 |
| SCGGAAGY_V$ELK1_02 | AATF; ACTB; ACYP1; AIP; AKT1S1; ALDOA; AMZ2; ANKS3; AP2S1; AP4M1; APTX; ARFGAP1; ARFIP2; ARPC2; ARPC4; ASB6; B3GALT6; B3GAT3; BANF1; BIN3; BLM; BZW2; C12orf57; C14orf119; C19orf47; C1orf122; CAP1; CBX8; CCDC103; CCDC85B; CCT7; CD2BP2; CDC123; CDC37; CDC45; CDCA3; CEP55; CHMP2A; CKS1B; CNOT10; CNPY3; COG4; COMMD5; COMMD6; COPE; COPS7B; COX17; COX5B; COX6A1; COX6B1; COX8A; CPSF3; CSNK2B; CWC15; CXXC1; DCLRE1C; DCTN5; DDOST; DDX49; DGUOK; DIABLO; DLX4; DNAJC7; DNTTIP1; DPCD; DPH2; E2F4; EBNA1BP2; EDC4; EEF1B2; EFNA5; EFTUD2; EIF1AD; EIF3H; EIF4A1; EIF5A; EME1; EMG1; ERCC1; ERH; EXOSC3; EXOSC5; FAM104A; FAM192A; FANCD2; FBXW9; FIBP; FKBPL; FOXH1; GAPDH; GAR1; GNL2; GPN2; GRWD1; GTF2A2; HARS; HNRNPD; IFI30; ING2; INO80E; ITGB1BP1; ITPA; JAGN1; KIF4A; KIF9; KLHDC3; KLHL17; KRTCAP2; KTI12; LAS1L; LIMD2; LLPH; LSM4; LSM5; LYPLA2; MAPRE1; MCM7; MCRS1; MCTS1; MEA1; MED8; MEF2B; METTL5; MFSD5; MORN2; MRPL27; MRPL33; MRPL43; MRPL52; MRPS18A; MRPS21; MTA2; MYL6B; NAGK; NASP; NDUFAF3; NECAP2; NEDD8; NFKBIB; NOC2L; NOL12; NOL7; NOSIP; NPRL2; NUDC; NUP37; NUTF2; ODF2; OTUB1; OVCA2; PABPC4; PARL; PARS2; PCGF1; PDAP1; PDCD6; PDZD11; PFDN6; PIH1D2; PLK4; PLP2; PMM2; POLR1C; POLR2F; POLR2H; POLR2K; POMP; PPAN; PPIL1; PPP4C; PRDX5; PRELID1; PRPF19; PSMA4; PSMA5; PSMA6; PSMB1; PSMB7; PSMC1; PSMC4; PSMD13; PTRH2; PUF60; PUS1; RAB24; RAD23A; RAVER1; RBCK1; RBM22; RFC2; RFC4; RFXANK; RNF181; RNPS1; ROMO1; RPF1; RPL11; RPL19; RPL26; RPL27; RPL31; RPL32; RPL36AL; RPL37; RPL37A; RPL6; RPLP2; RPS14; RPS18; RPS19BP1; RPS25; RPS3; RPS3A; RPS5; RPS6; RRAS; SART1; SDF2; SDHAF2; SEC13; SEMA4A; SF3A3; SF3B4; SH3GLB2; SHKBP1; SIPA1; SIRT6; SLC25A14; SLC35C2; SMUG1; SNF8; SNRPB; SNRPE; SPATA17; SPSB2; SRP14; SRP19; SSBP1; SSU72; STOML2; STX4; STXBP2; SUMO1; SUPT5H; TADA3; TAF10; TAF11; TBC1D13; TCOF1; THAP11; TIMM10; TMEM101; TMEM186; TMEM199; TMEM208; TMEM222; TOMM22; TOMM40; TPX2; TRADD; TRAPPC1; TRAPPC4; TRIM11; TRIM46; TRMT1; TRMT112; TUFM; TXNDC12; U2AF2; UBE2F; UBE2Z; UBL5; UBXN1; UFC1; UNC13D; UQCRH; URM1; USE1; USF1; UXT; VPS16; WDR34; WDR46; WDR73; WDR74; WDR83; WFDC3; WRAP53; XAB2; YARS; YKT6; YTHDF2; YWHAE; ZBTB8OS; ZNF394; ZNF408; ZNF580; ZNF668 |
| KMCATNNWGGA_UNKNOWN | ADAMTSL1; AP1G1; APOLD1; ARHGAP29; ARNTL; BCL2L13; CGGBP1; CRAT; CUX1; DLG2; DMD; GBF1; HMGXB4; ING3; MAP4K3; NR2F2; PPP4R2; PTCH1; RUNX1T1; SNX13; SP3; SPRED2; STAG1; TBC1D14; TMED10; TSHZ1; UBR3; WASL; ZBTB11; ZNF281; ZNF41 |
| YNGTTNNNATT_UNKNOWN | ACADSB; AMFR; AR; ASPA; ATP2A2; CALD1; CHD2; CHD6; CHN2; CLMN; CPEB4; CTNND1; DAAM1; DCUN1D1; DIS3L; DLC1; DMD; DOCK4; DSG1; DUSP10; EMP1; FMR1; FOXN3; HIPK1; IKZF5; ITGA1; ITPR1; JMJD1C; KDM6A; KIF13A; KLF9; MAGI1; MIA2; MXI1; MYOCD; NECAP1; NEDD4; NFIX; NUMB; PAN2; PGRMC1; PIK3R1; POFUT1; PPP2R5A; PPP2R5E; R3HDM2; RAPH1; RSF1; RUNX1T1; S1PR1; SEC24D; SH2D4A; SLC33A1; SMARCA2; SORBS2; SOX5; SP4; STAG2; STARD13; TAB2; TAL1; TCF12; THRB; THSD4; TOB1; TSC22D2; WDTC1; ZBTB37; ZFPM2; ZNF148; ZNF521; ZNF641 |

**Supplementary Table 7. Analysis of the relationship between PPIH levels and clinicopathological features of LIHC.**

| Pathologic features | Group | Serum PPIH content(pg/ml) | t-value | P-value |
| --- | --- | --- | --- | --- |
| Age | ＜58y | 232.11±12.45 | 0.558 | 0.586 |
|  | ≥58y | 228.52±13.27 |  |  |
| Gender | Male | 228.27±12.93 | 1.401 | 0.183 |
|  | Female | 239.20±6.00 |  |  |
| AFP | ＜7ng/ml | 238.35±8.46 | 1.987 | 0.068 |
|  | ≥7ng/ml | 225.72±11.51 |  |  |

**Supplementary Table 8. Analysis of the relationship between PPIH levels and clinicopathological features of COAD.**

| Pathologic features | Group | Serum PPIH content(pg/ml) | t-value | P-value |
| --- | --- | --- | --- | --- |
| Age | ＜50 | 222.49±11.74 | 0.478 | 0.640 |
|  | ≥50 | 219.54±12.57 |  |  |
| Gender | Male | 220.35±12.93 | 0.232 | 0.820 |
|  | Female | 221.89±10.56 |  |  |
| CEA | ＜5ng/ml | 223.73±11.37 | 1.506 | 0.154 |
|  | ≥5ng/ml | 214.45±11.57 |  |  |
| CA19-9 | ＜27.5ng/ml | 220.52±14.04 | 0.151 | 0.882 |
|  | ≥27.5ng/ml | 221.52±6.11 |  |  |
| Lymph node metastases | Yes | 218.38±10.48 | 1.849 | 0.086 |
|  | No | 231.46±13.96 |  |  |
| TNM staging | Ⅰ-Ⅱ | 231.25±14.29 | 1.803 | 0.093 |
|  | Ⅲ-Ⅳ | 218.43±10.47 |  |  |

**Supplementary Table 9. Analysis of the relationship between PPIH levels and clinicopathological features of GC.**

| Pathologic features | Group | Serum PPIH content(pg/ml) | t-value | P-value |
| --- | --- | --- | --- | --- |
| Age | ＜58.5 | 232.35±12.68 | 0.555 | 0.588 |
|  | ≥58.5 | 228.27±16.48 |  |  |
| Gender | Male | 229.98±15.56 | 0.184 | 0.857 |
|  | Female | 231.73±9.54 |  |  |
| CEA | ＜5ng/ml | 230.27±14.63 | 0.230 | 0.982 |
|  | ≥5ng/ml | 230.49±16.18 |  |  |
| CA19-9 | ＜27.5ng/ml | 231.61±14.03 | 0.745 | 0.469 |
|  | ≥27.5ng/ml | 224.66±17.52 |  |  |
| Lymph node metastases | Yes | 229.40±14.98 | 0.512 | 0.617 |
|  | No | 234.224±13.10 |  |  |
| TNM staging | Ⅰ-Ⅱ | 223.98±4.64 | 0.804 | 0.436 |
|  | Ⅲ-Ⅳ | 232.65±14.73 |  |  |

**Supplementary Table 10. Analysis of the relationship between PPIH levels and clinicopathological features of BC.**

| Pathologic features | Group | Serum PPIH content(pg/ml) | t-value | P-value |
| --- | --- | --- | --- | --- |
| Age | ＜47.5 | 220.75±12.71 | 1.196 | 0.252 |
|  | ≥47.5 | 229.58±16.55 |  |  |
| Lymph node metastases | Yes | 226.33±13.91 | 0.159 | 0.876 |
|  | No | 224.46±28.80 |  |  |
| TNM staging | Ⅰ-Ⅱ | 227.24±15.16 | 0.270 | 0.792 |
|  | Ⅲ-Ⅳ | 225.07±15.86 |  |  |
