# Supplementary material for: PPIH acts as a potential predictive biomarker for patients with common solid tumors
Source: BMC Cancer. 2024 Jun 4;24:681. doi: 10.1186/s12885-024-12446-9 (PMC11151604; doi:10.1186/s12885-024-12446-9)

**Supplementary Figure 1, related to Figure 1D. Western blotting analysis of PPIH protein expression in HCC and matched liver normal tissue.**


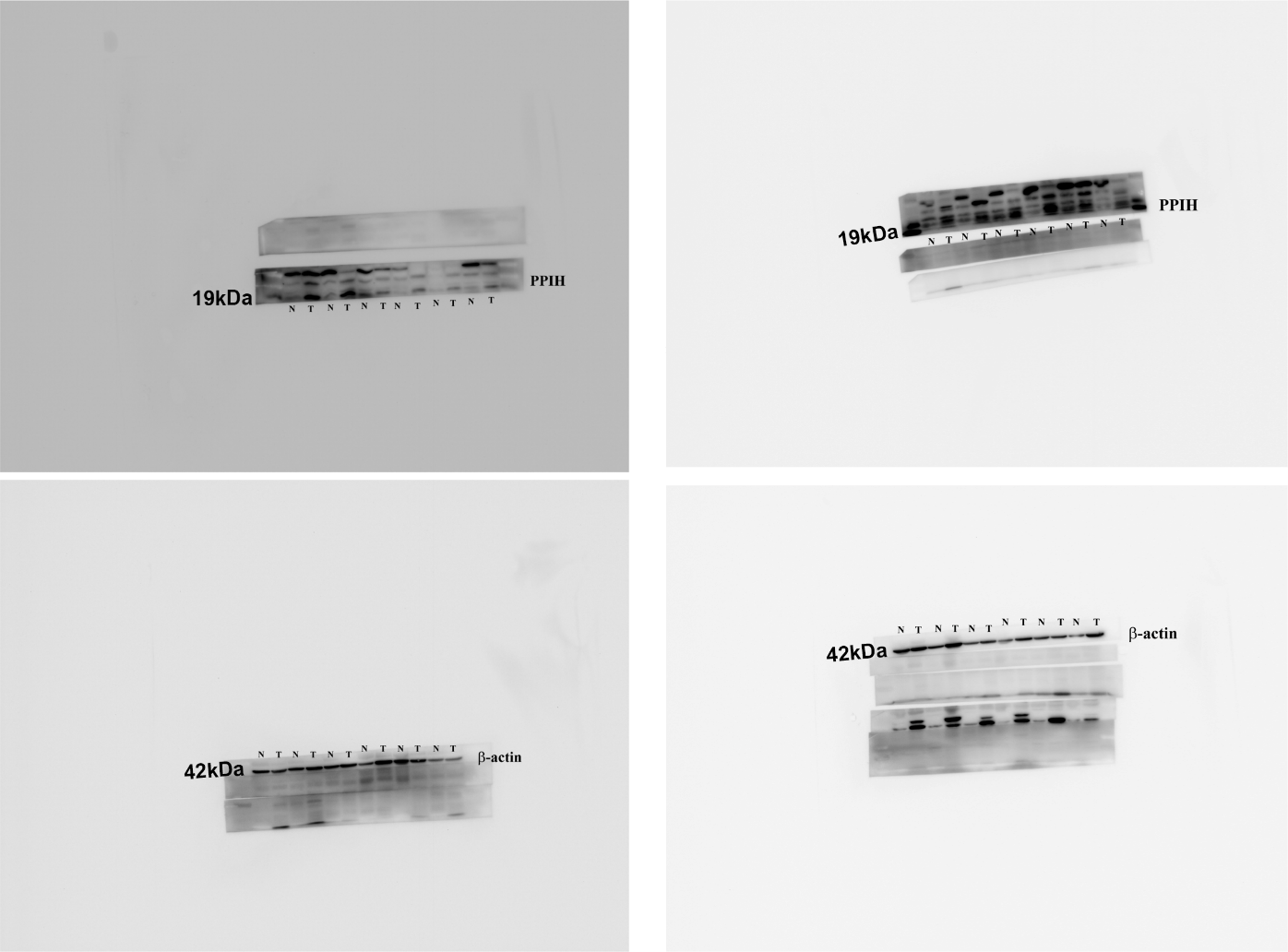

Supplement: Supplementary file 2 — Supplementary Material 2 [file 12885_2024_12446_MOESM2_ESM.docx]
